# A Computational Investigation of Four Sesquiterpene [4+2] Trimers, Inubritantrimers A–D, and Their Synthetic Intermediates Isolated from *Inula britannica* L.

**DOI:** 10.3390/molecules31101759

**Published:** 2026-05-20

**Authors:** Xiaoyun Xia, Xiandong Du, Zhifeng Chen, Sisi Yu, Chaojie Wang

**Affiliations:** School of Pharmaceutical Sciences, Wenzhou Medical University, Wenzhou 325035, China; xiaxiaoyun@wmu.edu.cn (X.X.); duxiandong@wmu.edu.cn (X.D.); zfchen@wmu.edu.cn (Z.C.); ssyu@wmu.edu.cn (S.Y.)

**Keywords:** *Inula britannica*, DFT, *exo*/*endo* configuration, molecular docking, molecular dynamics simulation

## Abstract

Triple-negative breast cancer (TNBC) is a clinically aggressive malignancy with extremely limited effective targeted therapies. Natural products are promising alternatives for anticancer drug discovery, whereas integrated computational approaches serve as efficient tools for novel lead identification. Herein, four novel spiro-polycyclic sesquiterpene [4+2] trimers (Inubritantrimers A–D) and eight synthetic derivatives from *Inula britannica* L. were investigated via DFT calculations at the ωB97xD/6-311++G(2d,p) level (for geometric, electronic, spectral, and reactivity parameters), network pharmacology, molecular docking against seven core breast cancer-related targets, 500 ns all-atom molecular dynamics (MD) simulation, and MM/PBSA analysis. The results showed that the *endo*-type cycloaddition products had superior structural stability, with all reactions thermodynamically spontaneous (ΔG < 0). Compound **11** exhibited the most potent and balanced binding activity, with a docking free energy of −13.45 kcal/mol to MTOR; MD and MM/PBSA confirmed stable complex formation (total binding free energy −21.13 kcal/mol), driven predominantly by hydrophobic interactions. This study first established a comprehensive stereochemistry–electronic structure–property–activity relationship for this rare sesquiterpene trimer class and identified compound **11** as a promising MTOR-targeted TNBC lead. It provided a theoretical basis for developing high-efficiency, low-toxicity natural anticancer agents.

## 1. Introduction

As an important branch of terpenoid natural products, sesquiterpenoids are C15 skeleton molecules composed of three isoprene units, which are widely distributed in plants, fungi, lichens, insects, and marine organisms [1]. Among them, [4+2] sesquiterpene adducts [2] have become a research hotspot in natural product chemistry and medicinal chemistry in recent years. *Inula britannica* L. belongs to the genus *Inula* of the Asteraceae family, and its dried capitulum has been officially recorded in the *Chinese Pharmacopoeia* (2020 Edition) [3]. It possesses various pharmacological effects, including resolving phlegm, relieving asthma, lowering adverse qi, arresting vomiting, soothing the liver, dredging collaterals, and promoting water metabolism [4]. In recent years, with advances in separation technologies and structural elucidation methods, researchers have discovered a series of sesquiterpenoids from this plant, including [4+2] cycloaddition polymers [5,6,7,8,9,10,11]. It was not until 2018 that the existence of sesquiterpene [4+2] trimers was confirmed [12]. The structure of such trimers is extremely rare, and the stereoconfiguration (*exo*/*endo*) is the core factor determining their druggability potential. However, there is currently no systematic study elucidating how the *exo*/*endo* stereoconfiguration regulates the electronic structure, physicochemical properties, druggability, and antitumor activities of these trimers. The lack of structure-activity relationships (SAR) to guide structural optimization limits the clinical translation of these natural products.

Studies have found that sesquiterpene adducts isolated from this genus exhibit potential cytotoxicity against breast cancer cells [13], which is of great significance for breast cancer treatment. In 2024, Tang’s team isolated four trimeric sesquiterpene [4+2] adducts with a spiro-polycyclic skeleton, named Inubritantrimers A–D, from *I. britannica* for the first time, and reported their moderate in vitro cytotoxic activities against breast cancer cells [14]. However, this study only completed structural identification and phenotypic screening. The underlying reasons for the differences in stability and activity among compounds with different configurations, the impact of stereoconfiguration on druggability and target binding, and the directions for their further rational optimization remain to be elucidated.

In this work, we systematically analyzed the structures, reaction mechanisms, biological activities, and spectral properties of Inubritantrimers A–D through theoretical calculations. We discovered the mechanism underlying differences in structural stability across types of sesquiterpene trimers and systematically established a complete stereochemistry-electronic structure-property-activity relationship for these trimers. By combining network pharmacology, molecular docking, and molecular dynamics, we revealed the core targets and molecular binding mechanisms of these compounds in breast cancer. Furthermore, we clarified the core pharmacophores and structural optimization strategies, providing systematic theoretical guidance for the rational design of such lead compounds.

## 2. Results and Discussion

### 2.1. Stereochemical Differences

#### 2.1.1. Geometric Structural Differences

**Figure 1:**
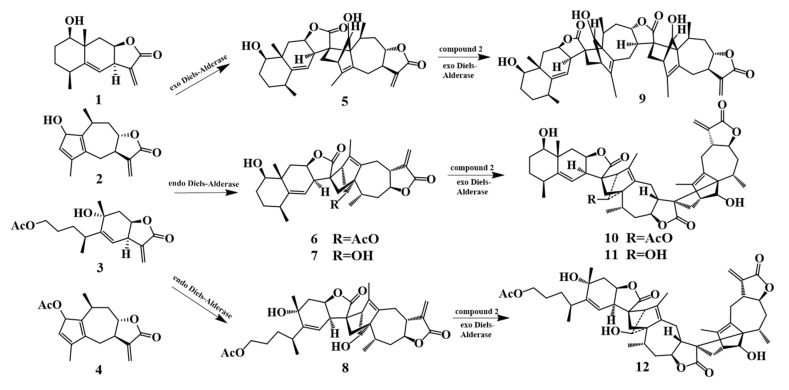
The 2D structural formulas of twelve compounds and their biosynthetic pathways.

**Figure 2:**
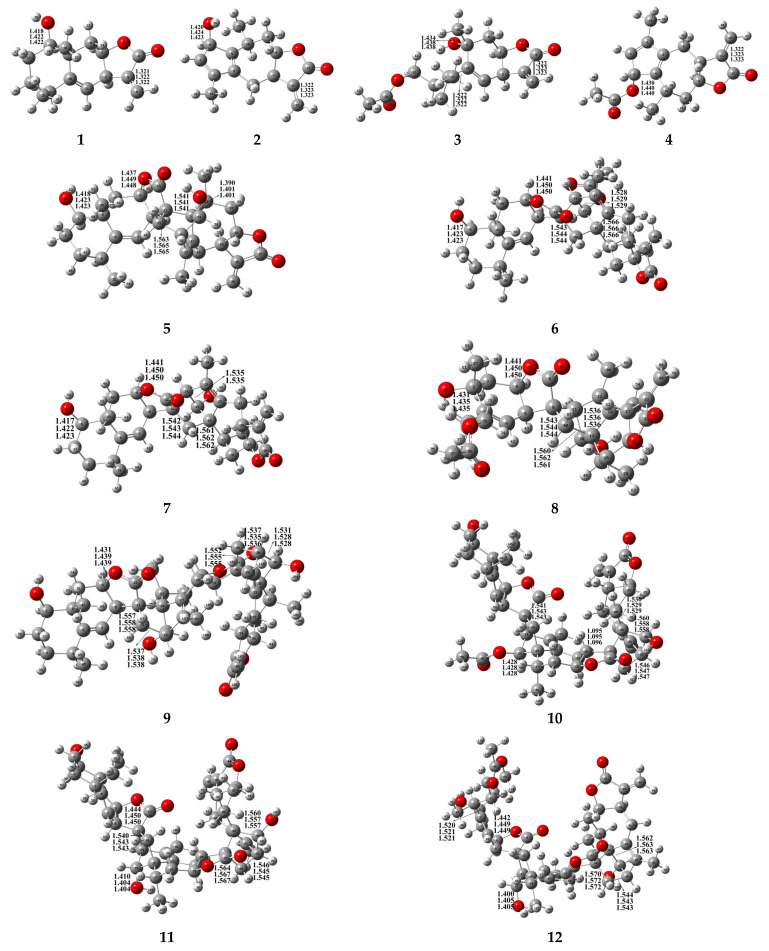
The optimized structures for twelve compounds in vacuum, water, and methanol environments calculated using ωB97xD/6-311++G(2d,p).

Figure 1 presents the 2D structural formulas of eight sesquiterpenoids and reactants isolated and identified from *I. britannica*. All twelve compounds were successfully optimized in vacuum, water, and methanol environments at the ωB97xD/6-311++G(2d,p) level of theory. The predominant stable conformations are shown in Figure 2, and key geometric parameters are detailed in Supplementary Table S1. Methodological validation results showed that the geometric parameters of the optimized compounds **5**, **6**, **10**, and **11** had an average absolute deviation of only 0.01 Å for key bond lengths and less than 1.1° for bond angles compared to the experimental values obtained from X-ray single-crystal diffraction. This indicates that the computational method employed in this study can accurately describe the geometric structures of these compounds, yielding highly reliable results.

In a vacuum, the C3=C6/C11=C16 double bonds of the four reactants 1–4 involved in the cycloaddition all have a bond length of 1.322 Å. After the completion of the cycloaddition reaction, these sites are converted into carbon–carbon single bonds, with bond lengths elongated by more than 0.2 Å. This perfectly aligns with the bond-length characteristics of carbon–carbon single bonds [15], confirming the rationality of the cycloaddition.

Comparing the newly formed C-C bonds in products with different configurations reveals that those in *exo*-configured products are generally longer. For instance, the C11–C15 bond formed in the *exo* compound **5** is 1.563 Å, whereas the corresponding C11–C39 bond in the *endo* compound **6** is only 1.543 Å. A shorter bond length corresponds to a higher bond order and greater bond energy. This result directly reveals that *endo* cycloaddition products possess superior intrinsic bonding stability. The electronic effects of substituents can significantly modulate molecular bond lengths and electron distribution: the C23–O32 bond length in compound **6** is 1.424 Å, while the C23–O33 bond length in compound **7** is 1.401 Å. This difference arises from the electronic effects of their attached functional groups: the hydroxyl group is electron-donating, whereas the acetoxy group [16] is electron-withdrawing, thereby affecting the electron density of the C-O bond. Solvent effects exert a minimal impact on the overall geometric structures of these compounds. The C=O bond lengths of all compounds in a vacuum are concentrated in the range of 1.400–1.450 Å. Polar solvents such as water and methanol cause a slight elongation of the C=O bond lengths by approximately 0.005–0.009 Å, without significant perturbation to the skeletal conformations of the molecules.

The stereoconfiguration and substituents jointly determine the bond angles, dihedral angles, and three-dimensional spatial conformations of the molecules, and these conformational differences directly affect the binding ability between the molecule and the target protein. A structural comparison between compounds **6** and **7** reveals that they differ only in the C23 substituent, yet they exhibit significant differences in the corresponding bond angles (113.4° vs. 114.5°) and dihedral angles (approaching 180° vs. −63.4°). Such conformational differences will directly alter the spatial orientation of molecular functional groups, thereby affecting spatial matching and interactions with the target protein’s binding pocket.

Skeletal analysis of trimeric compounds **9**–**12** shows that by analyzing the angles formed around the spiro carbons (C14/C16/C13) (i.e., C11–C14–C16/C11–C16–C14/C8–C13–C11), the angles in the *exo-exo* configuration compound **9** are significantly larger than those in the *endo-exo* configuration compounds **10** and **11**. Meanwhile, the bond angles at C38/C33/C30 confirm that compounds with the same addition mode exhibit no significant differences in bond angles at the core sites. Further analysis of the dihedral angles at C14/C16/C13 and C38/C33/C30 for compounds **9**–**12** reveals that this class of trimeric molecules adopts highly folded, non-planar conformations, which may result in high adaptability to spherical target pockets [17].

#### 2.1.2. Thermodynamic Property Evaluation

Chemical thermodynamics is the core theoretical tool for elucidating the spontaneity, stereoselectivity, and product stability of chemical reactions. Based on the fully optimized conformations at the ωB97xD/6-311++G(2d,p) level, vibrational frequency analysis and calculations of thermodynamic parameters for twelve compounds were completed under standard state conditions (298.15 K, 1 atm), obtaining core parameters including zero-point energy (ZPE), Gibbs free energy (G), enthalpy (H), entropy (S), and internal energy (U) (Table 1). These parameters were used to characterize the mechanism and intrinsic selectivity of the uncatalyzed Diels-Alder (D-A) reaction during the synthetic process. The reaction Gibbs free energy (ΔG) [18] was calculated according to formulas in the literature. ΔG < 0 indicates that the reaction is thermodynamically spontaneous at 298 K/1 atm; a larger absolute value of ΔG suggests a more thorough reaction and stronger spontaneity.

The results showed that the ΔG values for all monomer-to-dimer and dimer-to-trimer [4+2] cycloaddition reactions in this series were negative, indicating that the entire biosynthetic pathway is thermodynamically feasible and can proceed spontaneously. The *exo/endo* stereoconfiguration is the core factor determining the reaction driving force and product stability. Compared with compound **5**, compound **7** exhibits stronger reaction spontaneity under ambient gas-phase conditions, leading to the preferential formation of *endo* addition products, which confirms the higher proportion of *endo* sesquiterpene cycloaddition products in natural products. Compound **6** has the largest absolute value of ΔG and is highly spontaneous to form; however, the acetoxy group is prone to hydrolysis, which poses a metabolic instability defect. Regarding the thermodynamic analysis of the trimeric products **9**–**12**, the *endo-exo* addition compound **11** has a smaller ΔG and superior thermodynamic stability compared to the *exo-exo* addition compound **9**. In isolation experiments, compound **11** was obtained mainly as stable, colorless crystals in higher yield, providing a basis for synthetic feasibility and supporting its subsequent druggability evaluation. Substituent effects also significantly affect the thermodynamic behavior of the products. Compound **12** has the smallest absolute value of ΔG and a weak synthetic driving force, which is due to the steric hindrance introduced by multi-substituents such as hydroxyl and acetoxy groups, leading to poor stability and great synthetic difficulty.

### 2.2. Spectroscopic Property Analysis

#### 2.2.1. ^1^H NMR and ^13^C NMR Characterization

Following the completion of geometric structure optimization, the ^1^H NMR and ^13^C NMR chemical shifts of eight compounds were calculated using the gauge-including atomic orbital (GIAO) method at the ωB97xD/6-311+G(2d,p) theoretical level. The computed chemical shift values in chloroform solvent, along with the corresponding experimental measurements, are summarized in Table S2.

**Figure 3:**
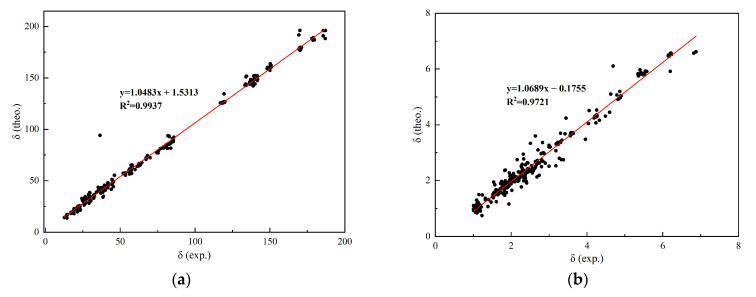
The theoretical and experimental ^13^C NMR (**a**) and ^1^H NMR (**b**) chemical shifts line up for compounds **5** to **12** computed with ωB97xD/6-311++G(2d,p). Outlier at C20 (δ_exp_. = 36.5 ppm, δ_theo_. = 94.1 ppm) arises from known GIAO limitations in highly congested bridgehead environments.

In the ^13^C NMR data, the theoretical chemical shift for the C23 position in compound **5** is calculated to be 151.7 ppm, whereas the experimental value is 134.4 ppm, indicating a notable discrepancy. A similar deviation was observed for the C20 carbon in compound **12** (theoretical: ~100 ppm vs. experimental: ~35 ppm), which stems from its extreme stereoelectronic environment, a well-documented limitation of the GIAO method for highly sterically hindered systems [19]. As a bridgehead carbon on the spirocyclic skeleton, C20 is tightly enclosed by three fused six-membered rings and an acetoxy group, causing significant electron cloud distortion that amplifies the calculation error. In contrast, the ^1^H NMR data exhibit excellent agreement between theory and experiment, with no significant deviations observed. For example, the theoretical chemical shift of H47 in compound **7** is 1.50 ppm, closely matching the experimentally determined value of 1.52 ppm. Linear regression analysis was performed on the computed and experimental NMR data for compounds **5**–**12**, and the results are presented in Figure 3. The correlation equation for ^13^C NMR is y = 1.0483x + 1.5313 (R^2^= 0.9937), and for ^1^H NMR it is y = 1.0689x − 0.1755 (R^2^= 0.9721), where y denotes the theoretical values and x represents the experimental measurements. The coefficient of determination (R^2^) reflects the strength of the linear relationship between predicted and observed values. All R^2^ values exceed 0.95, demonstrating high predictive accuracy of the computational approach and confirming the reliability of the ωB97xD/6-311++G(2d,p) method for structural prediction of [4+2] cycloaddition-derived sesquiterpenoids. Moreover, the superior fit observed for ^13^C NMR compared to ^1^H NMR indicates that this method achieves higher simulation precision for carbon resonance assignments than for proton spectra.

#### 2.2.2. Infrared Absorption Spectroscopy

**Figure 4:**
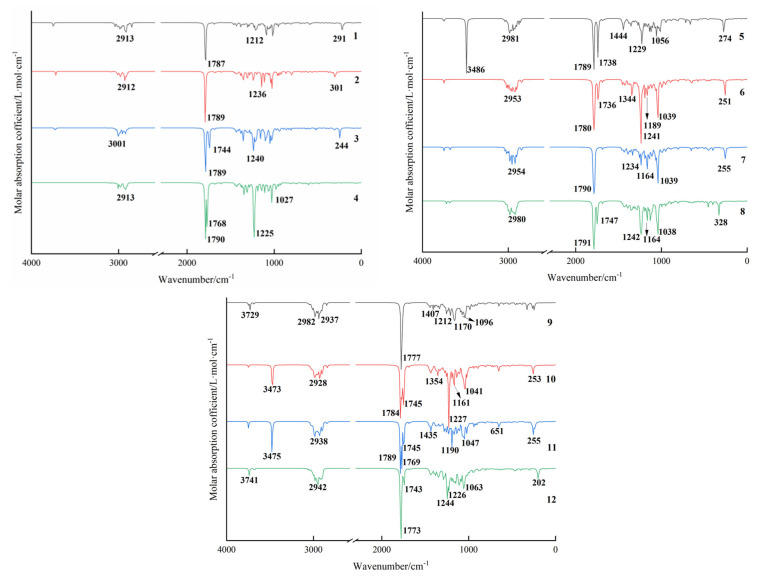
The IR absorption spectra for twelve compounds in vacuum computed with ωB97xD/6-311++G(2d,p).

Infrared absorption spectroscopy is a powerful analytical technique for characterizing molecular functional groups, bonding features, and spatial conformations. Density functional theory offers an efficient and computationally economical approach for predicting the vibrational spectra of organic molecules. Figure 4 presents the calculated infrared absorption spectra of twelve compounds in a vacuum environment at the ωB97xD/6-311++G(2d,p) level of theory. Table S3 summarizes the theoretical and experimental assignments of key infrared characteristic absorption peaks for compounds **6**, **7**, and **9**–**12**, along with their corresponding peak intensities. The theoretically calculated vibrational frequencies are based on the harmonic oscillator model, which inherently introduces systematic deviations from the fundamental transitions of real molecular anharmonic vibrations. To improve the accuracy of the calculated results and ensure comparability between computed and experimental data, a scaling factor of 0.957 was applied, as recommended by the Computational Chemistry Comparison and Benchmark Database, to account for systematic deviations arising from anharmonicity and basis-set limitations.

The absorption band in the 3400–3500 cm^−1^ region is assigned to the O–H stretching vibration, while multiple absorption bands in the 2900–3000 cm^−1^ range are attributed to C–H stretching vibrations. All compounds exhibit a strong characteristic absorption peak of C=O stretching vibration near 1750 cm^−1^ [20], a characteristic absorption feature of the C=C double bond around 1685 cm^−1^, and absorption bands below 1000 cm^−1^ are assigned to in-plane bending vibrations of C–H bonds.

Notably, compound **5**, the carbonyl group (C=O) forms an intramolecular hydrogen bond with the adjacent hydroxyl group (–OH), resulting in a redshift and pronounced intensification of the O–H stretching absorption band [21]. Meanwhile, compared to compound **6**, compound **5** shows a downshifted C=O stretching signal from the acetoxy group at 1736 cm^−1^. A comparative analysis of compounds **10** and **11** reveals that the acetoxy group in compound **11** experiences reduced steric hindrance and greater vibrational freedom, leading to a slightly lower C=O stretching frequency.

A comparative analysis of the spectra of the *exo* and *endo* configuration products reveals that only minor differences exist in the vibrational frequencies of the C=O and C–C bonds adjacent to the cycloaddition site between the two isomers. The *exo* and *endo* isomers derived from this type of [4+2] cycloaddition only differ in the spatial orientation of the chiral centers, with completely identical types, numbers, and bonding modes of functional groups. Thus, the intrinsic vibrational frequencies of the core functional groups are not significantly affected.

Discrepancies between experimentally measured infrared vibrational frequencies and theoretical values may arise from the combined effects of several factors, including intramolecular hydrogen-bond formation, intermolecular association, and influences of experimental conditions [22]. Furthermore, inherent limitations exist within the computational methodology: the theoretically calculated frequencies are derived from the harmonic oscillator approximation, whereas real molecular systems exhibit anharmonic behavior [23,24]. Overall, the theoretically calculated spectra show strong overall agreement with the experimentally measured data, while minor deviations may be observed for the specific frequencies of individual vibrational modes.

#### 2.2.3. Ultraviolet–Visible Absorption Spectrum

Ultraviolet–visible absorption spectroscopy provides insight into the electronic structure, energy level distribution, and chemical stability of molecular systems. The ωB97xD functional, which incorporates dispersion and long-range corrections, enables accurate computation of UV absorption spectra for organic molecules and delivers reliable spectral predictions [25,26]. All calculations were performed in methanol at the ωB97xD/6-311++G(2d,p) theoretical level. The full-wavelength UV-Vis absorption spectra of twelve compounds are presented in Figure S1, and the excited state parameters and assignment of electronic transitions for the main absorption bands are detailed in Table 2.

The reaction precursors **1**–**4** exhibit more abundant absorption peaks (2-3 absorption bands). In contrast, the dimers and trimers (compounds **5**–**12**) formed via [4+2] cycloaddition show only one major absorption band in the range of 100 to 350 nm, with their maximum absorption wavelengths clustering around 200 nm, at the boundary between the far-UV region (150–200 nm) and the mid-UV region (200–300 nm). This phenomenon arises from the disruption of the conjugated diene system in precursor molecules by cycloaddition, which reduces the degree of conjugation in the products, leading to a blue shift and fewer absorption bands. This finding is fully consistent with the bonding characteristic of double bonds converting to single bonds identified in the aforementioned geometric structure analysis.

The maximum absorption peaks of compounds **6** and **7** are at 190.41 nm and 191.10 nm, respectively, with only a slight difference between them. The electronic effects of the acetoxy group and hydroxyl group have no obvious regulatory effect on the UV absorption characteristics of this class of compounds. When comparing the maximum absorption wavelengths of the *exo*/*endo* stereoisomers, no notable distinction is found between them. Changes in the spatial orientation of the chiral center do not alter the overall electronic energy level distribution of the molecule. Thus, the *exo*/*endo* stereoconfiguration of such compounds cannot be distinguished by the absorption characteristics in the UV functional group region, which provides clear methodological guidance for the spectral analysis of analogous natural products.

Analysis of excited-state transitions clarifies the electronic nature of UV absorption in this class of compounds. All electronic transitions governing the absorption characteristics are centered on π → π* transitions [27], and are directly associated with the conjugated systems of C=C double bonds and carbonyl groups in the molecules. The main absorption band of compound **11** derives from the electron transition from the HOMO-5 orbital to the LUMO, with a contribution rate of 91.4%. The S_0_ → S_5_ excited state contributes to the maximum absorption peaks of compounds **3**, **7,** and **10**–**12**, with the most prominent contribution to compound **11**. The S_0_ → S_3_ excited state is responsible for the UV absorption peaks of compounds **1**–**3**, and its contribution rate to compound **3** reaches 86.6%.

### 2.3. Electronic Structure and Chemical Reactivity

#### 2.3.1. Frontier Molecular Orbital Analysis

The reactivity of molecules can be effectively predicted using frontier molecular orbital (FMO) theory. The HOMO is defined as the orbital with the highest energy that is occupied by electrons. In contrast, the LUMO corresponds to the lowest-energy unoccupied orbital that can accept electrons. The spatial distribution patterns of HOMO and LUMO, along with their energy gap (Δ*ε* = E_HOMO_ − E_LUMO_), directly govern the chemical reactivity of compounds and their potential binding modes with biological targets [28]. In this study, frontier orbital analysis was performed on twelve compounds using density functional theory at the ωB97xD/6-311++G(2d,p) level. Table 3 summarizes the calculated HOMO and LUMO energies, as well as the corresponding energy gaps, for all compounds under a vacuum environment. The spatial distributions of these frontier orbitals are illustrated in Figure S2.

The Diels-Alder (D-A) cycloaddition reaction is the core step in the biosynthesis of this class of sesquiterpene trimers, and frontier orbital interactions are the fundamental factors governing its feasibility and stereoselectivity. For D-A reactions with normal electron demand, the core driving force arises from the effective orbital overlap between the HOMO of conjugated dienes and the LUMO of dienophiles [29,30]. In this study, compounds **1** and **3** act as dienophiles while compounds **2**, **4**–**8** function as dienes. The orbital energy level matching between these two types of reactants is in full accordance with the rules of frontier orbital interaction for D-A reactions, which provides direct verification for the chemical rationality of this biosynthetic pathway from the perspective of electronic structure.

Among compounds **5**–**12**, the energy required for the electronic transition of the *exo*-configuration is relatively lower than that of the *endo*-configuration. This leads to a faster reaction rate for the *exo* pathway, whereas the endo pathway yields more stable products. This result aligns perfectly with the experimental observation that *endo*-configuration products account for a higher proportion in natural products.

The LUMO energy levels of compounds **5** and **9** are markedly lower than those of other compounds in the same series, a consequence of the strong intramolecular hydrogen bonds formed between the carbonyl oxygen and adjacent hydroxyl groups within the molecule. This interaction can effectively lower the LUMO energy level of dienophiles and enhance the orbital overlap efficiency between the HOMO of dienes and the LUMO of dienophiles, thereby significantly facilitating the progression of the D-A cycloaddition reaction. It forms a complete evidence chain together with the regulatory effects of intramolecular hydrogen bonds identified in the aforementioned infrared spectroscopy and thermodynamic analysis. Compound **9** has the smallest energy gap and thus exhibits favorable reactivity, with its HOMO and LUMO energy distributions both localized at the spiro ring at the C14 position. The acetoxy-containing substituents attached at the C2 position of compounds **8** and **12** exert no influence on the energy distribution. A direct comparison between compounds **5** and **6** shows that hydroxyl substitution is a stronger electron donor than the acetoxy group, with a correspondingly lower HOMO energy level.

In the transition from dimers to trimers, the energy distribution shifts toward the newly incorporated moiety—compound **2**—and the effects induced by hydroxyl and acetoxy groups are negligible. This indicates that the degree of polymerization primarily affects the spatial distribution of orbitals rather than the overall reactivity of the molecule, thus providing a clear boundary for the structural optimization of trimer derivatives: the target-binding ability can be modulated by modifying the newly introduced structural moieties, without altering the core reactivity of the molecule itself.

#### 2.3.2. Molecular Surface Electrostatic Potential Maps (MSESPMs)

**Figure 5:**
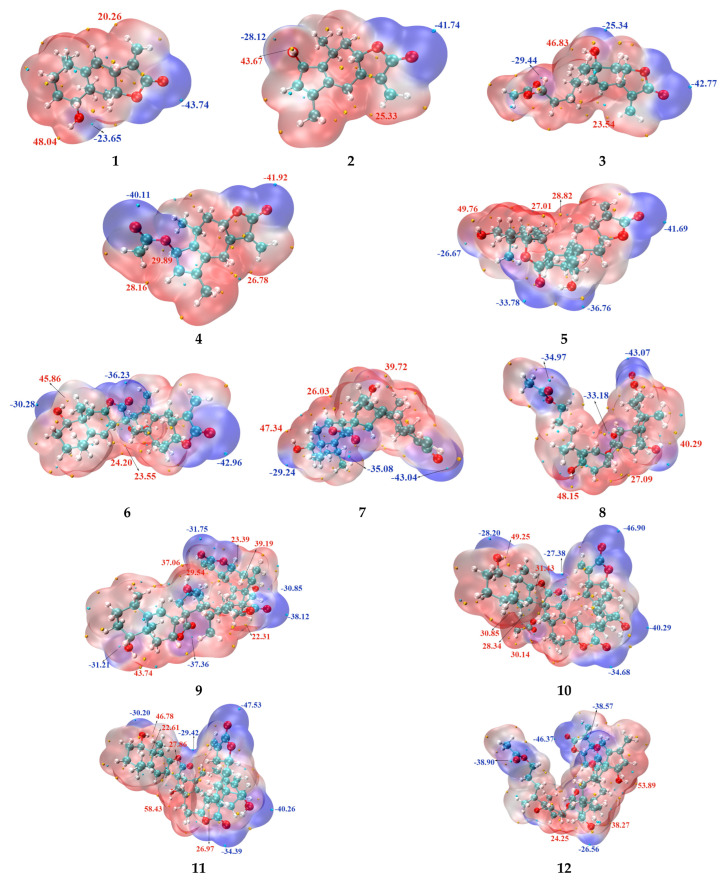
The electrostatic potential maps for twelve compounds under a vacuum environment using the ωB97xD/6-311++G(2d,p) method.

The molecular electrostatic potential (MEP) is a physical property that reflects the distribution of electron density and serves as an effective tool for visualizing and quantifying surface charge characteristics at the molecular level [26]. In this study, MEP maps for twelve compounds under a vacuum environment were calculated using Multiwfn and visualized with VMD. The resulting figures illustrate the spatial distribution of electrostatic potential across the molecular surfaces and the locations of extreme values. Red regions denote positive electrostatic potentials (electron-deficient sites), while blue regions indicate negative potentials (electron-rich sites). Yellow spheres mark local maxima, corresponding to regions of highest positive potential, and cyan spheres represent minima, or areas of most negative potential (Figure 5).

Analysis reveals that the maximum electrostatic potential values for the twelve compounds range from 20.26 to 58.43 kcal/mol, while the minimum values range from −47.53 to −23.65 kcal/mol. The most negative potential regions are predominantly localized around carbonyl oxygen atoms, followed by hydroxyl oxygen atoms—sites characterized by high electron density and thus prone to electrophilic attack. Conversely, hydrogen atoms attached to hydroxyl groups typically exhibit pronounced positive potentials, forming distinct electrophilic regions with low electron density that are susceptible to nucleophilic interactions. The hydroxyl hydrogen atom at the C23 position of compound **6** bears an extremely strong positive potential and serves as a highly active nucleophilic reaction site. For compounds **7** and **8**, where the same position is substituted by an acetoxy group, the range and intensity of negative potential in the vicinity of this substituent rise remarkably, a direct consequence of the strong electron-withdrawing inductive effect of the acetoxy group. Under the influence of the electron-withdrawing effect from adjacent carbonyl and hydroxyl groups, the C3=C6 double bond of compound **1** and the conjugated double bond region of compound **2** both display positive potential characteristics, which further enrich the reactive sites of the molecules.

The *exo* versus *endo* stereoconfiguration significantly modulates the molecular electrostatic potential (MEP) surface distribution. In compound **5** (*exo* isomer), an intramolecular hydrogen bond facilitates charge delocalization, resulting in an expanded region of negative electrostatic potential—thereby enhancing its electrophilic reactivity relative to compound **7** (*endo* isomer), which lacks this stabilizing interaction. In contrast, compound **7** with the *endo* configuration fails to form such intramolecular hydrogen bonds, and its electrostatic potential distribution tends to be more localized. For other positions, no significant differences in MEP distribution are observed between *exo* and *endo* configurations. Figure S3 presents a histogram of the electrostatic potential surface areas. Comparative evaluation of the twelve molecules shows that their total molecular surface areas range from 270 to 720 Å, with surface potential values widely distributed, primarily clustered within the −15 to 30 kcal/mol range. Table S4 provides quantitative data on molecular surface properties, classifying surface regions into negative potential zones (<−10 kcal/mol) and positive potential zones (>10 kcal/mol). Statistical analysis indicates that the surface area fraction of negative-potential regions is generally smaller than that of positive-potential regions, suggesting an overall hydrophobic character and compatibility with hydrophobic binding pockets in target proteins.

The introduction of acetoxy groups significantly increases the molecular surface area and raises the proportion of negative-potential regions. A comparison between compounds **11** and **12** demonstrates that the presence of an acetoxy group reduces the positive potential intensity near adjacent hydroxyl functionalities. Relative to compound **10**, which exhibits a strong negative potential only at C23 with limited spatial influence, compound **12** exerts a broader electronic effect, generating a localized positive potential band near C27. This feature enhances hydrophobic interactions and promotes stacking interactions with adjacent hydrophobic residues within protein binding sites [31].

#### 2.3.3. Global Reactivity Descriptors

Based on the calculated HOMO and LUMO energies, this study derived global reactivity descriptors for twelve compounds, summarized in Table 4. These parameters provide quantitative insights into molecular stability, electron-donating/accepting tendencies, and chemical reactivity trends. As shown in Table 4, under three different environments—vacuum, water, and methanol—the ionization potentials of the compounds range from 7.87 to 9.01 eV. At the same time, all electron affinities are negative, indicating weak electron binding and limited electron-uptake capacity across the series [32]. Except for compounds **3** and **10**–**12**, the IP values are highest in vacuum and lowest in water. Among the sesquiterpenoid derivatives (compounds **5**–**12**), compound **5** exhibits the highest IP value, whereas compound **12** has the lowest. Due to solvation effects, the IP values of compounds **5**, **7**–**10** reach their minimum in vacuum, while those of the remaining compounds attain maximum values under the same environment.

In the context of cycloaddition reactivity prediction, analysis of reactants **1**–**4** reveals that compounds **3** and **4** exhibit elevated EA values due to the presence of acetoxy groups, reflecting enhanced electron-accepting capacity with respect to chemical potential. Compounds **1** and **3** exhibit relatively low μ values (−4.33 eV and −4.41 eV, respectively) and high η values (4.60 eV and 4.59 eV), suggesting greater thermodynamic stability and a propensity to attract electrons [33]. Compounds **2** and **4** exhibit relatively large S values and low ω values, rendering them highly prone to electron transfer. Their reactivity characteristics align perfectly with the core operational mechanism of Diels-Alder reactions with normal electron demand—that is, dienes donating electrons and dienophiles accepting electrons. This alignment affirms the feasibility of the proposed reaction pathway at the level of global reactivity. A direct comparison of the relevant values reveals that compounds **2** and **3** exhibit notably higher reactivity. Furthermore, compound **4** contains an acetoxy group, resulting in a large dipole moment, significant charge separation, and pronounced molecular polarity. It is well established that when the diene bears an electron-donating group (e.g., –OH) and the dienophile features a conjugated electron-withdrawing group (e.g., >C=O), the *endo* adduct is typically favored [34].

The dipole moment is a fundamental physical quantity that quantifies the spatial separation between positive and negative charge centers within a molecule and serves as a key metric for assessing molecular polarity [35]. Comparison of compounds **5**–**8** shows that the *exo*-configured compound **5** possesses a larger dipole moment, which is significantly amplified in polar solvents. Although its softness is comparable to that of other isomers, it exhibits higher electronegativity and electrophilicity indices, indicating stronger electron affinity and greater interaction potential with electrophilic species. Compounds **6** and **7** differ only in the substitution of acetoxy and hydroxyl groups, with no significant variation in their global reactivity. This demonstrates that such substituents merely modulate electronic effects at local sites and exert only a negligible influence on the molecule’s overall reactivity. A comparison of compounds **9** to **12** shows that the *exo*-*exo* configuration features a relatively small dipole moment and enhanced hydrophobicity for the molecule as a whole, along with a slightly lower absolute hardness (*η*) and a higher electrophilic index (*ω*), thus enabling favorable binding to the hydrophobic targets of proteins. For the *endo*-*exo* configurational compounds, the acetoxy group attached to the terminal of the C1 side chain in compound **12** has a small dipole moment and low EA and *χ* values, resulting in weak electron-accepting capacity; this endows the compound with superior potential for binding to hydrophilic protein targets.

#### 2.3.4. Local Reactivity Descriptor

The maximum values of local reactivity descriptors serve as effective indicators for identifying atomic sites within a molecule that are more susceptible to nucleophilic or electrophilic attack compared to other positions [36]. In this study, the local reactivity of twelve compounds under a vacuum environment was systematically analyzed using Fukui functions (*f*^+^, *f*^−^, *f*^0^), local softness (*s*^+^, *s*^−^, *s*^0^), electrophilicity indices (*ω*^+^, *ω*^−^, *ω*^0^), and Mulliken charge values. The resulting data are summarized in Table 5. A higher *f*^+^ value at a given site indicates enhanced nucleophilic activity, whereas a larger *f*^−^ value reflects greater electrophilic character [37].

This study provides a comprehensive atomic-level elucidation of the regional selectivity mechanism underlying this class of [4+2] ring addition reactions. Analysis of reaction precursors **1**–**4** demonstrates that the maxima of both *f^−^* and *f*^+^ are localized at the C6/C11 double bond, indicating extensive electron delocalization across the conjugated system and the absence of well-defined, localized electron-rich or electron-deficient regions, and that, combined with Mulliken charge analysis [38], compounds **1** and **3** exhibit a pronounced positive charge at the C6 position, rendering this site more favorable for electrophilic attack. In contrast, compounds **2** and **4** display elevated *f*^+^ values at the C1 atom within the conjugated diene framework, where reduced electron density increases susceptibility to nucleophilic attack. According to the regioselectivity principles of the D-A reaction, these complementary charge distributions between reacting centers promote efficient bond formation.

In compounds **5**–**8**, compound **5** (*exo* configuration) exhibits *f*^−^ and *f*^+^ maxima at the C26 carbon atom due to the influence of an extended π-conjugated system. For *endo*-configured compounds **6**–**8**, the primary reactive sites remain largely unaffected by acetoxy or hydroxyl substituents: the *f*^−^ maximum is located at the carbonyl carbon atoms C12/C9, identifying them as preferred sites for electrophilic attack; meanwhile, the *f*^+^ maximum occurs at the C26/C23 carbonyl carbon atoms, suggesting their role as potential nucleophilic attack sites. Notably, the absolute Mulliken charge values at these active sites are greater in the *endo* isomers than in their *exo* counterparts, indicating increased electron deficiency and thus a higher propensity toward nucleophilic reactions. In the *exo-exo*-configured compound **9**, both *f*^−^ and *f*^+^ maxima converge at C41. With a Mulliken charge of +0.064 a.u., this site demonstrates a strong tendency for nucleophilic reactivity. For compounds **11** and **12**, the *f*^−^ maximum occurs at the same atomic position; however, compound **11** exhibits a higher *f*^−^ value and a lower atomic charge magnitude, indicating superior electron-accepting capability and relatively higher chemical reactivity.

These reactivity trends can be fully rationalized by the intrinsic structural features of the scaffold: the carbonyl group serves as both the core hydrogen-bonding site and the reactive center, and modifying substituents near the carbonyl group allows researchers to regulate electronic density at the binding site, thereby enhancing hydrogen-bonding affinity with target proteins. At the same time, the *exo/endo* stereoconfiguration directly determines the distribution and activity of the reaction sites. Designers can select advantageous conformational frameworks based on specific requirements: the *endo* configuration is more effective for optimizing metabolic stability, while the *exo* configuration provides better control over reaction activity.

### 2.4. Pharmacodynamic Evaluation

The absorption (A), distribution (D), metabolism (M), excretion (E), toxicity (T), and physicochemical properties of compounds are critical determinants in the drug discovery process. It has been widely reported that approximately 60% of candidate drugs fail in clinical trials due to unfavorable ADMET profiles [39]. To address this challenge, a variety of computational tools, both online and offline, are now available to support ADMET prediction and evaluation. In this study, a systematic assessment of the pharmacodynamic behavior, biological activity, and toxicity of twelve compounds was conducted using three established predictive platforms: SwissADME, ACD/Percepta, and ADMETLab 3.0.

Among the eighteen freely accessible web-based platforms capable of predicting physicochemical and pharmacodynamic parameters, ADMETLab is recognized for offering the broadest range of predictions and high predictive accuracy, making it one of the most comprehensive tools currently available [39]. However, due to the relatively high molecular weights of compounds **9**–**12** (MW > 500), which exceed the upper size limit supported by the SwissADME platform, reliable predictions could not be generated for these compounds. As a result, comparative analysis was restricted to the results obtained from ACD/Percepta and ADMETLab 3.0, with detailed data summarized in Table 6.

Reactivity, an indicator of a compound’s potential to act as a reactive species, was evaluated to assess chemical stability. Compounds **1**–**4** exhibit higher reactivity scores, indicating a greater propensity for chemical transformations. In contrast, among compounds **5**–**8**, compound **8** displays a lower reactivity score, indicating enhanced chemical inertness and improved stability under physiological conditions. For compounds **9**–**12**, elevated molecular weights may impair gastrointestinal dissolution and absorption, limiting oral bioavailability. Concurrently, their low LogS values reflect poor aqueous solubility, further hindering efficient oral absorption. In comparison, compounds **6**–**8** within the series **5**–**12** demonstrate relatively favorable solubility profiles. Furthermore, compounds **10**–**12** exhibit high LogP values, indicative of strong lipophilicity, which may enhance membrane permeability but also raise concerns regarding potential issues with metabolic stability, clearance, and off-target toxicity.

The subsequent ADMET pharmacological property predictions were primarily focused on compounds **5**–**12**. Drug-likeness was assessed using Lipinski’s rule of five, combined with prediction results from the ACD/Percepta and ADMETLab 3.0 platforms. Compounds **5** and **7** demonstrated higher drug development potential, whereas compounds **10** and **12** exhibited lower drug-likeness, indicating limited prospects for further development.

P-glycoprotein (P-gp) is a critical efflux transporter that actively pumps xenobiotics out of intestinal epithelial cells, thereby reducing oral bioavailability. Despite this, predicted human intestinal absorption (HIA) values indicate that all twelve compounds possess high intestinal permeability, suggesting favorable absorption characteristics. Caco-2 permeability data further reveal that compound 6 exhibits the highest transcellular transport capacity, which may facilitate rapid tissue distribution and efficient delivery to target sites, such as tumor tissue.

Oral bioavailability (HOB, F > 50%) was evaluated using the ADMETLab 3.0 platform. The results show that the predicted bioavailability for all compounds falls below the established threshold, implying suboptimal systemic exposure. This limitation could be addressed through formulation strategies or structural modifications to enhance absorption and metabolic stability. For patients with triple-negative breast cancer (TNBC) who have developed brain metastases, blood–brain barrier (BBB) penetration is a key determinant of central nervous system (CNS)-related adverse effects. Predictions indicate that compounds **8** and **11** exhibit relatively low BBB permeability, suggesting reduced risk of CNS toxicity and an improved safety profile. Additionally, plasma protein binding (PPB) levels for compounds **5**–**12** are generally high, which may restrict the concentration of free, pharmacologically active drug, thereby narrowing the therapeutic index.

With respect to cytochrome P450 enzyme inhibition, none of the compounds showed significant inhibitory activity against CYP1A2, CYP2C9, CYP2C19, CYP3A4, or CYP2D6, indicating a low potential for clinically relevant drug–drug interactions. Metabolic stability assessments in human liver microsomes (HLM) revealed that compound **7** exhibits the best stability, while compound **9** has an instability probability approaching 100%, suggesting rapid metabolic degradation. Moderate clearance (Cl) values are conducive to maintaining steady-state plasma concentrations; except for compound **12**, all other compounds display clearance within a moderate range. Notably, eight of the compounds are predicted to have short half-lives (T_1/2_), which may necessitate frequent dosing regimens to sustain therapeutic efficacy in clinical settings.

Toxicity predictions revealed discrepancies between platforms: the ACD/Percepta platform classified compounds **5**–**12** as having low genotoxic potential (Ames), indicating initial safety, whereas ADMETlab 3.0 predicted a high risk of genetic toxicity, highlighting inter-platform variability and model-dependent uncertainty. Cardiovascular safety assessment indicated minimal human Ether-à-go-go-Related Gene (hERG) channel inhibition across most compounds, suggesting a low risk of QT prolongation and arrhythmogenesis. However, a high probability of drug-induced liver injury (DILI) was predicted for nearly all compounds, representing a significant safety concern. In neurotoxicity analysis, compound **11** showed exceptionally low drug-induced neurotoxicity (Neurotoxicity-DI), indicating a favorable neurological safety profile. Rat oral acute toxicity (ROA) predictions further indicated that compounds **7** and **8** have lower toxicity scores, suggesting manageable acute toxicity risks.

Overall, integrating physicochemical properties and ADMET profiles, compounds **5**–**9** and **11** demonstrate promising pharmacodynamic behavior and acceptable safety margins, warranting further pharmacological investigation. Structure-activity relationship (SAR) analysis suggests that the presence of electron-withdrawing groups, such as acetoxy substituents, is associated with increased toxicity. Therefore, future structural optimization should aim to reduce or replace these moieties to improve overall safety and tolerability. Hepatic metabolic stability plays a pivotal role in determining in vivo exposure: compound **7** (*endo* configuration) exhibits superior metabolic stability in HLM compared to compound **5** (*exo* configuration). Similarly, *endo*-*exo*-configured compounds demonstrate greater metabolic stability than their *exo*-*exo* configuration. It must be emphasized that these conclusions are derived from computational models and represent theoretical predictions under idealized conditions. Given the differences in algorithms and training datasets among software platforms, variations in predictive outcomes are expected. Moreover, the complexity and heterogeneity of human physiology necessitate experimental validation through in vitro assays and in vivo studies before any definitive conclusions can be drawn.

### 2.5. Network Pharmacology Analysis

#### 2.5.1. Target Prediction

**Figure 6:**
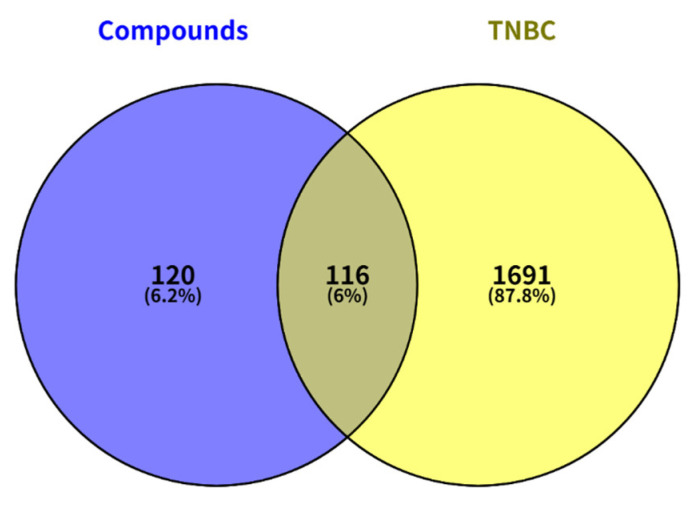
Venn diagram of chemical component targets and TNBC-related targets.

**Figure 7:**
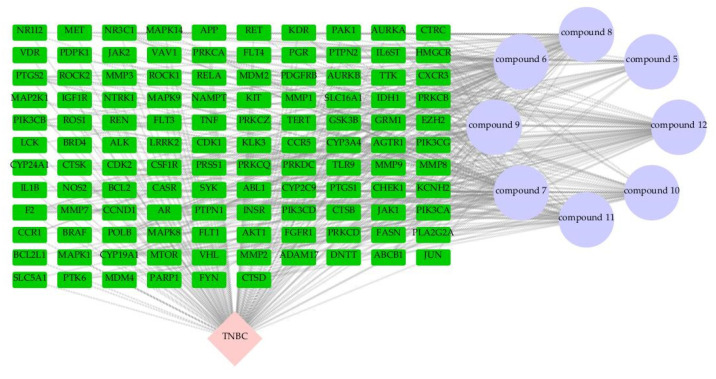
Drug–component–target network diagram.

The targets of each component were integrated, and duplicates were removed, resulting in a total of 236 potential targets associated with the chemical constituents. Using a threshold of relevance > 20, TNBC-related targets were screened from the GeneCards database. The GeneCards “relevance score” is a composite metric that integrates the quantitative strength of gene–disease associations, the breadth of literature support, and the quality of experimental evidence [40]. A threshold of >20 was applied to prioritize high-confidence triple-negative breast cancer (TNBC)-associated targets while filtering out low-relevance annotations—aligning with established network pharmacology conventions for target prioritization. These were then combined with targets from three additional disease databases, yielding 1,807 targets related to triple-negative breast cancer. Mapping the compound-related targets against the disease-related targets identified 116 overlapping targets, suggesting that these may represent the key points of action for the compounds in TNBC treatment (Figure 6). These targets were further visualized using Cytoscape 3.10.4 software [41], creating a network diagram of the drug-component-target relationship (Figure 7).

#### 2.5.2. Construction of the PPI Network for Common Targets

**Figure 8:**
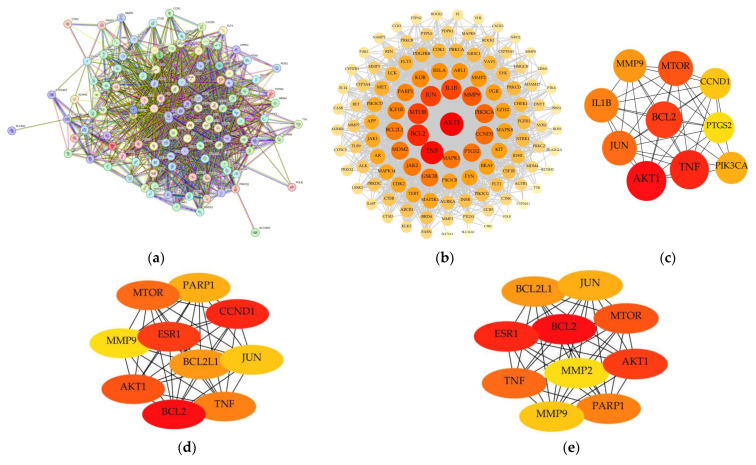
Construction and visualization of the common target PPI network. (**a**) PPI network obtained from the String database; (**b**) PPI network visualized by Cytoscape; (**c**) core target PPI network; (**d**) protein–protein interaction (PPI) network of core targets, filtered by relevance score > 15; (**e**) protein–protein interaction (PPI) network of core targets, filtered by relevance score > 25.

The intersecting targets from the Venn diagram were imported into the String database, yielding a PPI network with 116 protein nodes and 1700 edges (Figure 8a) [42]. In this network, nodes represent proteins, and edges indicate interactions between them. Different colors correspond to distinct types of evidence supporting these interactions, including experimental data, text mining, database annotations, gene neighborhood, gene fusion, and gene co-expression. The network was further visualized using Cytoscape 3.8.2 (Figure 8b), where larger node size and darker color indicate greater importance of the protein within the interaction network. Using the Cyto-Hubba plugin, the top 10 targets were selected as core targets based on their degree values (Figure 8c). This study suggests that key targets involved in the anti-TNBC activity of these compounds include AKT1, BCL2, TNF, and MTOR.

To assess the robustness of the target identification strategy with respect to the relevance score threshold, we conducted sensitivity analyses using alternative cutoffs of 15 and 25. Both analyses yielded highly consistent results: 153 overlapping targets were retained at the >15 threshold, and 110 at the >25 threshold—critically, the four core hub targets (AKT1, BCL2, TNF, and mTOR) remained invariant across all three threshold conditions (i.e., the original threshold and the two sensitivity thresholds). The protein–protein interaction (PPI) networks constructed from the top 10 hub targets under each cutoff (>15 and >25) are shown in Figure 8d and Figure 8e, respectively.

#### 2.5.3. GO and KEGG Enrichment Analyses

**Figure 9:**
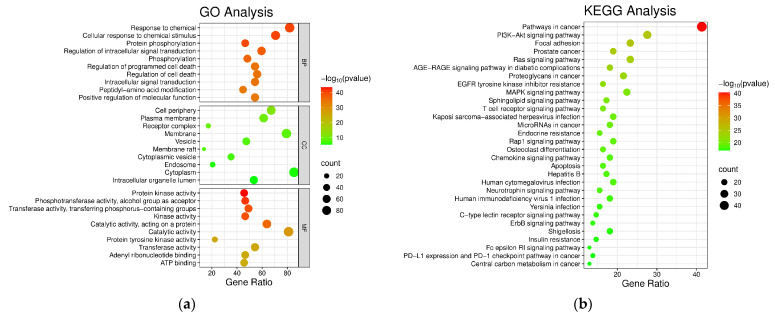
GO and KEGG analyses of common targets: (**a**) an enrichment bubble plot depicting GO analysis; (**b**) an enrichment bubble plot showing KEGG analysis.

To investigate the potential molecular mechanisms underlying the therapeutic effects of these chemical constituents against triple-negative breast cancer (TNBC), the overlapping targets were subjected to Gene Ontology (GO) enrichment analysis in the DAVID database [43], with a particular focus on the biological processes and molecular functions associated with TNBC. A total of 1576 GO terms were identified, including 1386 Biological Process (BP) terms, 85 Cellular Component (CC) terms, and 109 Molecular Function (MF) terms [41]. The top 10 terms in each category, ranked by *p*-value, were selected for visualization (Figure 9a).

In addition, 172 Kyoto Encyclopedia of Genes and Genomes (KEGG) pathways were significantly enriched. The top 30 enriched pathways were visualized using a bubble plot, in which the x-axis represents the enrichment level of target genes, bubble size indicates the number of enriched genes, and color intensity corresponds to the *p*-value (Figure 9b). The GO and KEGG enrichment results collectively suggest that the anti-TNBC activity of this class of sesquiterpene [4+2] trimers is not mediated by a single target but rather by the coordinated regulation of multiple targets involved in key oncogenic pathways, particularly the PI3K-Akt signaling pathway, thereby influencing cell proliferation and apoptosis. These findings provide a plausible mechanistic explanation for the in vitro cytotoxic activity reported by Tang et al. [14], address the limitation of previous studies in which bioactivity was observed but the underlying mechanism remained unclear, and offer a mechanistic basis for the rational design of subsequent lead compounds.

### 2.6. Molecular Docking Validation

#### 2.6.1. Screening of Docking Proteins

Two scientific considerations guided target selection. First, characteristic driver targets across different molecular subtypes of breast cancer were included to align with the reported in vitro and in vivo cytotoxicity data. Second, the selected targets were integrated with the core regulatory targets identified in the preceding network pharmacology analysis, thereby ensuring both rigor and relevance in target selection. Accordingly, the targets were classified into two categories:

1. Cell subtype-specific target matching:

Previous experimental studies have demonstrated that compounds **5**–**9** and **11** exhibit therapeutic potential against breast cancer. Except for compound **7**, the remaining compounds showed varying degrees of cytotoxic activity against three breast cancer cell lines, namely MDA-MB-231, MDA-MB-468, and MCF-7 [13,14]. Owing to differences in molecular subtype, these cell lines are associated with distinct therapeutic targets.

The GeneCards platform (http://www.genecards.org) was used to retrieve genes associated with the three cell lines above [44]. In addition, literature evidence indicates that EGFR and TP53 are the most characteristic alterations in MDA-MB-468 cells and among the most frequently investigated targets in current preclinical studies [45]. For MCF-7 cells, ESR1 is the key gene. It serves as a central target in hormone-dependent breast cancer, with most related studies focusing on its interactions with ligands such as tamoxifen and phytoestrogens [46]. MDA-MB-231 is the most commonly used highly invasive TNBC cell model, and investigations of its associated targets, together with molecular docking analyses, are of considerable importance in drug development. In 2022, one study reported that compound **6** significantly inhibited the growth of MDA-MB-231 tumors in mice by 85% [5]. Under the same experimental conditions, compound **6** also completely blocked the proliferation of MCF-7 cells in nude mice without causing obvious toxicity. In both models, compound **6** was found to downregulate MDM2 expression in tumor tissues [47].

2. Core targets identified by network pharmacology:

Based on the node degree ranking in the PPI network, together with the screening results of the GO and KEGG enrichment analyses, the identified core targets represent key nodes through which this class of compounds may exert broad-spectrum anti-breast cancer effects. Specifically, AKT1 and MTOR are central components of the PI3K-Akt signaling pathway [48], which was identified by KEGG enrichment analysis as the most significantly enriched core pathway associated with anti-breast cancer activity and is also critically involved in regulating tumor proliferation and apoptosis. BCL2 is a core anti-apoptotic protein in the apoptosis pathway [49] and was identified as a key gene in the GO term “regulation of cell apoptosis,” indicating its direct relevance to tumor cell survival. TNF, as a central mediator of tumor microenvironment regulation and inflammatory responses, plays an important role in tumor proliferation, invasion, and immune evasion [50].

The three-dimensional crystal structures of all selected targets were downloaded from the RCSB PDB database (https://www.rcsb.org), including EGFR (PDB: 1M17), ESR1 (PDB: 7UJ8), MDM2 (PDB: 4ERE), AKT1 (PDB: 3O96), MTOR (PDB: 4DRH), BCL2 (PDB: 4AQ3), and TNF (PDB: 2AZ5). These proteins were subsequently subjected to molecular docking with the pharmacologically relevant screened compounds **5**–**9** and **11**, with tamoxifen included as the positive control.

#### 2.6.2. Analysis of Docking Results

**Figure 10:**
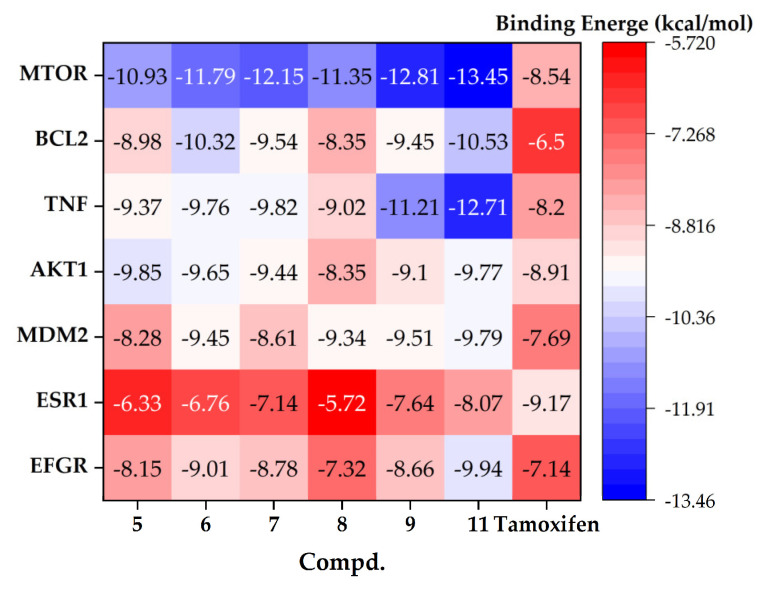
Heatmap of molecular docking binding free energy between target compounds and seven core anti-breast cancer targets (Tamoxifen as positive control).

The binding free energy was used as the primary indicator to evaluate the binding affinity between the compounds and their targets. In general, a binding affinity lower than −6 kcal/mol is considered indicative of good target binding. The heatmap of the binding energies between the six compounds and the seven targets is presented in Figure 10. Tamoxifen, a first-line clinical anti-breast cancer drug, was used as the positive control to assess the target-binding capacities of these compounds systematically.

ESR1 is the direct target of clinical endocrine therapeutic agents such as tamoxifen. Overall, the binding affinities of this series of compounds toward ESR1 were weaker than that of the positive control tamoxifen (−9.17 kcal/mol), but all compounds still exhibited good binding activity. Among them, compound **11** showed the strongest binding activity (−8.07 kcal/mol), with the smallest activity gap relative to tamoxifen. All compounds exhibited excellent binding activity toward EGFR, with binding free energies below −7.0 kcal/mol, markedly better than that of the positive control, tamoxifen (−8.07 kcal/mol). All compounds also exhibited strong binding activity toward MDM2, with binding free energies below −8.0 kcal/mol. Among them, compounds **6**, **9**, and **11** showed the most pronounced binding activities, all markedly superior to tamoxifen (−7.69 kcal/mol), thereby providing an atomic-level explanation for the molecular mechanism by which compound **6** downregulates MDM2 expression.

For the four core targets, AKT1, MTOR, BCL2, and TNF, identified within anti-breast cancer signaling pathways via network pharmacology, all evaluated compounds exhibited significantly stronger predicted binding affinities compared to those observed for non-core, cell−level targets. Although tamoxifen was initially included as a broad clinical reference to contextualize the overall anti-breast cancer potential of these natural products [51,52], we acknowledge that target-specific, clinically approved or investigational inhibitors constitute more pharmacologically relevant benchmarks, particularly for non-ESR1 targets. Accordingly, we extended our molecular docking and binding free energy analysis by incorporating three rigorously validated clinical inhibitors, using identical computational protocols (force field parameters, solvation model, sampling strategy, and post-processing methodology) as in the original study. For the PI3K–Akt pathway core targets, all compounds exhibited predicted binding free energies of <−8.0 kcal/mol toward AKT1 and <−10.0 kcal/mol toward MTOR. Notably, compound **11** displayed the strongest predicted affinity for MTOR (−11.24 kcal/mol), closely approaching that of the clinical gold-standard inhibitor rapamycin (−11.47 kcal/mol) [53]; its affinity for AKT1 (−9.86 kcal/mol) was also comparable to that of the clinical-stage allosteric inhibitor MK−2206 (−10.13 kcal/mol) [54]. Comprehensive comparative binding free energy data are summarized in Table 7. These results collectively support direct, high-affinity engagement of AKT1 and mTOR by the compounds, thereby substantiating their capacity to modulate the PI3K–Akt pathway and exert on-target antitumor effects. BCL2 and TNF, key regulators of intrinsic apoptosis and tumor microenvironment remodeling, respectively, were identified as core functional targets through Gene Ontology (GO) enrichment analysis. Among all tested compounds, compound **11** demonstrated superior binding performance against both targets: −10.53 kcal/mol for BCL2 (marginally exceeding venetoclax, −10.31 kcal/mol) [55] and −12.71 kcal/mol for TNF. These findings provide computational evidence that this compound class exerts anti-breast cancer activity through dual modulation of apoptotic signaling and immune–stromal crosstalk within the tumor microenvironment.

Overall, this series of sesquiterpene oligomers exhibited excellent binding activities toward all seven core anti-breast cancer targets. Compound **11** showed the most balanced and strongest binding activity across all seven targets. In addition, it possessed favorable chemical stability, drug-likeness, and reactivity, indicating that it is the most promising anti-breast cancer lead compound in this series. Compounds **6** and **9** exhibited prominent binding to specific targets, consistent with their in vitro and in vivo antitumor activities, and may therefore serve as alternative lead compounds.

**Figure 11:**
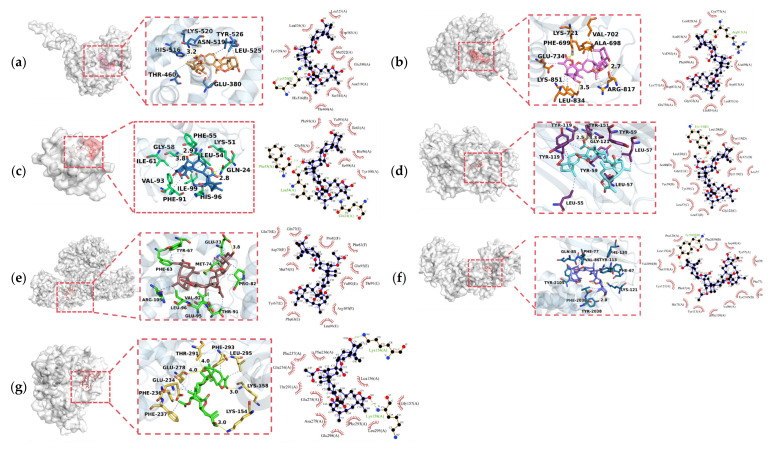
Molecular docking binding modes of compound **11** with seven key target proteins: (**a**) ESR1; (**b**) EGFR; (**c**) MDM2; (**d**) TNF; (**e**) BCL2; (**f**) MTOR; (**g**) AKT1.

For compounds **6**, **9**, and **11**, the interactions with the target proteins were further analyzed, and both their hydrogen-bonding and hydrophobic interactions are summarized in Table 8. The 3D and 2D binding modes of compound **11** are shown in Figure 11, while those of the other compounds are presented in Figure S4. The yellow solid lines represent hydrogen-bonding interactions, the blue dashed lines indicate hydrophobic interactions, and the green lines denote salt bridges. Compared with sesquiterpene dimers, the trimers exhibited relatively higher sensitivity toward breast cancer-related targets.

Statistical analysis of the non-covalent interactions revealed that the number of residues involved in hydrophobic interactions was 2- to 6-fold higher than that of hydrogen-bonding residues across all compound-target complexes, with hydrophobic residues accounting for over 80% of total interacting residues for kinase targets (MTOR and EGFR). This demonstrated that hydrophobic interactions are the dominant stabilizing force for all complexes, with hydrogen bonds serving only as auxiliary anchors for target recognition.

The bulky, rigid hydrophobic core of the sesquiterpene [4+2] trimers provides the essential binding scaffold, with hydrophobic and van der Waals interactions acting as the primary driving force for target binding. Hydrogen bonds, formed between the compounds’ hydroxyl/acetoxy carbonyl groups and polar pocket residues, are few in number but critical for target selectivity. Compound **11** (dihydroxyl-substituted) displayed the strongest MTOR binding affinity, driven by extensive hydrophobic stacking with 10 key hydrophobic residues in the MTOR ATP-binding pocket (including PHE67, VAL86, TYR113, PHE2039, TYR2105)—all highly conserved residues essential for MTOR kinase activity.

Notably, compound **9** (*exo-exo* configuration) showed a markedly different interaction profile from compound **11** (*endo-exo* configuration), confirming that *exo*/*endo* stereochemistry tunes target binding and selectivity by modifying the compounds’ spatial conformation and binding pocket complementarity.

### 2.7. Molecular Dynamics Simulation

Based on the docking results, the MTOR-compound **11** complex, which exhibited the best binding performance, was selected for a 500 ns all-atom molecular dynamics simulation to investigate its interaction mechanism further.

**Figure 12:**
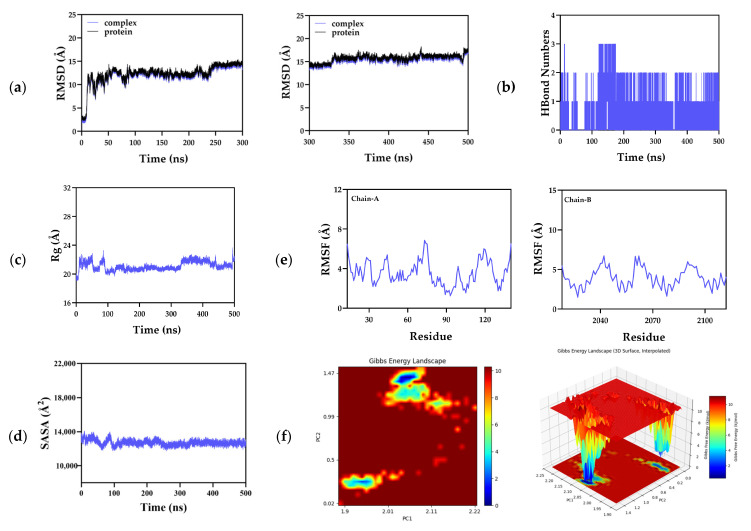
(**a**) RMSD values for apo MTOR and the MTOR–compound **11** complex; (**b**) hydrogen-bonding interactions between compound **11** and MTOR; (**c**) Rg values for compound **11** in complex with MTOR; (**d**) SASA values of compound **11** and MTOR; (**e**) RMSF values for compound **11** in relation to MTOR; (**f**) free energy landscape of the complex.

Root-mean-square deviation (RMSD) is a useful indicator of the conformational stability of both proteins and ligands. It reflects the extent to which atomic positions deviate from their initial coordinates. Smaller deviations indicate better conformational stability. Therefore, RMSD was used to assess the equilibration of the simulation system. As shown in Figure 12a, the RMSD of the complex increased sharply during the first 10 ns of the simulation, indicating significant initial conformational relaxation of the system. From 10 to 330 ns, the fluctuation amplitude decreased, the upward trend in RMSD became more gradual, and the value remained within the 12–14 Å range. After 330 ns, the system reached equilibrium and stabilized at around 17 Å, suggesting that the complex had completed its conformational rearrangement and entered a relatively stable, relaxed state. From 10 to 330 ns, the RMSD fluctuation amplitude progressively diminished, the upward drift in RMSD slowed markedly, and values remained confined within 12–14 Å. Beyond 330 ns, the system attained structural equilibrium and stabilized at approximately 17 Å—indicating completion of conformational relaxation and entry into a thermodynamically stable, low-energy state. MTOR is a large, multidomain protein featuring a highly dynamic kinase domain [56]. To dissect the structural origin of the observed RMSD behavior, we conducted a parallel 500 ns all-atom molecular dynamics (MD) simulation of apo MTOR under identical computational conditions. As shown in Figure 12a, the RMSD trajectories of the MTOR–compound **11** complex and apo MTOR are nearly superimposable, demonstrating that ligand binding does not perturb the intrinsic conformational sampling of the kinase domain. This controlled comparative analysis confirms that the global RMSD fluctuations reflect MTOR’s inherent structural flexibility rather than ligand-induced distortions or complex destabilization.

Notably, two distinct conformational events were identified in the RMSD trajectories: (i) a stepwise increase from approximately 12 Å to 14–15 Å occurring at ~250 ns, and (ii) a transient spike to ~18 Å at ~440 ns, which rapidly decayed back to the 16–17 Å plateau. The stepwise transition at ~250 ns corresponds to an irreversible shift between two thermodynamically stable conformational substates of MTOR, mediated by large-scale rearrangements of its solvent-exposed loops and intrinsically disordered terminal regions [56]. In contrast, the transient spike at ~440 ns represents a reversible, sub-microsecond conformational fluctuation localized to these same dynamic domains—consistent with intrinsic thermal sampling rather than a persistent structural perturbation. Critically, the near-identical RMSD profiles of the MTOR–compound **11** complex and apo MTOR—under identical simulation conditions—demonstrate unequivocally that both events originate solely from MTOR’s intrinsic conformational landscape and are neither induced nor modulated by ligand binding, dissociation, or complex destabilization. These mechanistic insights have been integrated into the revised manuscript.

Hydrogen bonds play an important role in ligand-protein binding and are directly associated with binding affinity and target inhibitory activity. Throughout the 500 ns simulation, the instantaneous number of hydrogen bonds between compound **11** and MTOR fluctuated between 0 and 3, without abrupt changes. Among these, the binding conformation containing one hydrogen bond represented the dominant conformational state of the system. The number of hydrogen bonds formed between the small molecule and the target protein during the dynamic process is shown in Figure 12b. During the 500 ns simulation, the number of hydrogen bonds between compound **11** and MTOR ranged from 0 to 3. At equilibrium, the instantaneous number of direct hydrogen bonds in the complex was predominantly 0, followed by 1, with an average of 0.56 hydrogen bonds over the entire trajectory. This result is consistent with the initial molecular docking analysis, which suggested a binding mode dominated by hydrophobic interactions, with hydrogen bonds serving an auxiliary anchoring role.

The radius of gyration (Rg) can be used to describe changes in overall structure and to characterize a protein’s compactness. A continuous increase in Rg indicates protein unfolding and reduced compactness, whereas stabilization of Rg within a constant range suggests that the protein remains well-folded and globally stable. During the simulation, the complex exhibited relatively stable fluctuations, with the Rg value remaining essentially constant at around 21 Å. No obvious expansion or contraction was observed in the small-molecule-target protein complex throughout the simulation, providing a global structural basis for the complex’s stable binding (Figure 12c). The solvent-accessible surface area (SASA) is an indicator of a protein’s surface area. In this simulation, the SASA of the target protein-small molecule complex was calculated (Figure 12d). The results showed no significant change in the SASA of the complex after ligand binding, further indicating that the protein and small molecule can maintain relatively stable binding and that the ligand effectively occupies the hydrophobic pocket of the target protein.

Root mean square fluctuation (RMSF) reflects the flexibility of amino acid residues in a protein. As shown in Figure 12e, most RMSF values of the complex were below 7 Å, while the RMSF values of the key residues involved in hydrophobic interactions were generally below 4 Å. The free energy landscape (FEL) of the complex is shown in Figure 12f. A color map was used to represent different free energy values, where the X-axis corresponds to PC2 (root-mean-square deviation) in nanometers (nm) and the Y-axis to PC1 (radius of gyration), also in nanometers. In the figure, red regions indicate relatively high free energy, whereas blue regions indicate relatively low free energy. Only one concentrated low-free-energy basin was observed in the complex system, located in the region of PC1 = 2.135 and PC2 = 0.3, with no dispersed distribution of multiple free-energy basins. This indicates that, during the equilibrium stage, the system adopted a single thermodynamically dominant stable conformation without significant conformational transitions, consistent with the RMSD analysis, which shows that the system reached dynamic equilibrium after 330 ns.

**Figure 13:**
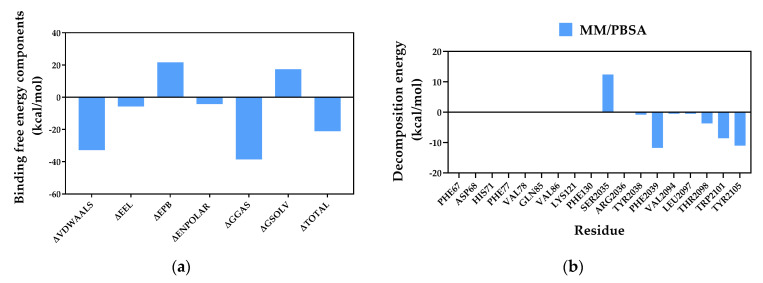
Binding free energy analysis of the compound **11** − MTOR complex via the MM/PBSA method. (**a**) Energy component decomposition of the total binding free energy; (**b**) residue − wise decomposition of the binding free energy contribution from MTOR amino acid residues.

Subsequently, based on the complex’s binding conformation, the thermodynamic binding affinity between compound **11** and MTOR was quantitatively evaluated. The binding free energy between the small molecule and the target protein was calculated using the MM/PBSA method, followed by residue-level energy decomposition analysis (Figure 13). The total binding free energy of the complex was −21.13 kcal/mol, indicating a very strong binding affinity of this molecule for the target protein, consistent with the results of static molecular docking. The van der Waals interaction energy (ΔVDWAALS = −32.81 kcal/mol) made the dominant contribution to the total binding free energy. Its absolute value was 5.7 times greater than that of the electrostatic interaction energy (ΔEEL = −5.75 kcal/mol), clearly demonstrating that hydrophobic interactions play a decisive role in the binding of compound **11** to MTOR.

In the complex system, residues PHE2039, TYR2105, TRP2101, THR2098, TYR2038, LEU2097, and VAL2094 exhibited relatively high energy-contribution values (Figure 13b). Among them, PHE2039, TRP2101, LEU2097, and VAL2094 are aromatic or aliphatic hydrophobic residues that mainly contribute to the binding free energy through van der Waals interactions and π–π stacking, serving as the major source of van der Waals energy. This is consistent with the conclusions drawn from the energy component analysis.

Taken together, the complex system exhibited stable binding, providing a theoretical basis for its use as a targeted-MTOR lead compound against TNBC.

## 3. Materials and Methods

### 3.1. DFT Calculations

Using the ωB97xD functional at the 6-311++G(2d,p) basis set level, the geometric and electronic structures of all compounds involved in the formation process shown in Figure 1 were optimized in a vacuum, and their infrared (IR) vibrational spectra were simultaneously calculated.

Based on the optimized structures, Inubritantrimers A–D (compounds **9**–**12**) were further optimized in simulated water and methanol solvent environments using the Conductor-like Polarizable Continuum Model (CPCM) within the Self-Consistent Reaction Field (SCRF) method. Subsequently, time-dependent density functional theory (TD-DFT) was employed to calculate the ultraviolet–visible (UV-Vis) absorption spectra in methanol and water. The obtained wavefunctions were used for electronic structure analysis and Conceptual Density Functional Theory (CDFT) index analysis. Global reactivity descriptors were calculated according to the formulas defined in references [57,58] to facilitate the prediction of molecular electrophilicity or nucleophilicity, including ionization potential (IP), electron affinity (EA), electronegativity (χ), global hardness (η), chemical potential (μ), global electrophilicity index (ω), and global softness (S). Concurrently, condensed Fukui functions were computed using the finite-difference approximation based on the atomic charges derived from natural population analysis (NPA), following established protocols described in the literature. These descriptors quantify site-specific nucleophilic and electrophilic reactivity within the molecular framework. All calculations and analyses were performed using Gaussian 16 [59], Multiwfn 3.8 [60], and VMD 1.9.3 [61] software.

### 3.2. Pharmacodynamic Simulation

The physicochemical properties (Lipinski’s Rule of Five) and pharmacodynamic characteristics (ADMET) of the compounds were systematically evaluated using ACD/Percepta 2012.2.0 software, the ADMETlab 3.0 platform [39], and the SwissADME platform [62] to screen for the most promising candidate compounds.

### 3.3. Network Pharmacology Calculation

#### 3.3.1. Target Protein Exploration

Based on the compounds’ structures, potential targets were predicted using the SwissTargetPrediction [63] and SEA databases. The collected targets were calibrated using the UniProt database (https://www.uniprot.org/) [64], after which non-human genes and invalid duplicate targets were removed to obtain standardized gene names. By entering the keyword “Triple Negative Breast Cancer” into the GeneCards [44], OMIM [65], and DisGeNET [66] databases, targets related to triple-negative breast cancer (TNBC) were retrieved. All targets from the three databases were integrated into an Excel spreadsheet, duplicate genes were removed, and the remaining genes were corrected using the UniProt database to obtain target gene information for TNBC.

The acquired drug targets and TNBC targets were pooled to screen for intersecting targets, and a Venn diagram was generated using an online platform (http://bioinfogp.cnb.csic.es/tools/venny/index.html; accessed on 16 January 2026) [42]. The shared gene targets were uploaded to the STRING database to construct a protein–protein interaction (PPI) network. The species was set to “Homo sapiens,” and the minimum required interaction score was set to 0.4 to ensure study reliability, with other parameters kept at their default settings. The results were saved in TSV format and imported into Cytoscape software for visualization [41]. The cytoHubba plugin was further used to evaluate the topological structure of the regulatory network and screen for core targets of the chemical constituents for treating TNBC.

#### 3.3.2. GO Enrichment and KEGG Pathway Analyses

The drug-disease intersection genes were uploaded to the DAVID database [43], with the gene identifier set to OFFICIAL_GENE_SYMBOL and the species to Homo sapiens. The DAVID 6.8 database was used to annotate the roles of the target proteins of the chemical constituents in treating TNBC across three Gene Ontology aspects: BP, CC, and MF. To elucidate the roles of these targets in signaling pathways, Kyoto Encyclopedia of Genes and Genomes pathway enrichment analysis was performed. The top 10 GO terms in BP, CC, and MF, along with the top 30 KEGG pathways, were selected as the primary gene function enrichment processes and signaling pathways for the chemical constituents used to treat TNBC, thereby predicting their underlying mechanisms of action.

#### 3.3.3. Molecular Docking and Molecular Dynamics Analysis

Molecular docking is a key computational method for evaluating the binding affinity and interactions between drug candidates and target proteins. To explore the binding interactions between the compounds and target proteins, we used PrankWeb 3 [67] to rapidly and accurately predict protein-ligand binding sites, and AutoDock 4.2.6 [68] for molecular docking simulations. Both protein and ligand structures were prepared in compatible formats: PDB files for proteins and MOL2 files for ligands, ensuring compatibility with the docking software. The docking results were analyzed for receptor-ligand interactions using the PLIP 2025 platform [69] and visualized with PyMOL 3.1.3 [70] and LigPlot+ 2.3.1 [71].

Molecular dynamics simulations were performed using GROMACS 2022 [72]. The receptor protein was parameterized using the AMBER99SB-ILDN force field (a refined variant of AMBER14SB with improved backbone and side-chain torsional potentials). In contrast, the small-molecule ligand was processed with ACPYPE (v2.2.0, based on Antechamber and acpype_1.0 dev3.1) to generate GAFF2-compatible topology files. Partial atomic charges were derived using the restrained electrostatic potential (RESP) method at the HF/6-31G* level of theory, ensuring quantum-mechanically informed, physically realistic charge distributions. The solvated system was constructed under cubic periodic boundary conditions, with a minimum solvent buffer distance of 1.0 nm between the protein–ligand complex and all box edges, employing the TIP3P water model for explicit solvation. Neutralization and physiological ionic strength (0.15 M NaCl) were achieved by replacing water molecules with Na^+^ and Cl^−^ ions using the gmx genion tool. Electrostatic interactions were treated with the particle mesh Ewald (PME) method (grid spacing < 0.12 nm; fourth-order B-spline interpolation; tolerance 1 × 10^−5^), with a real-space cutoff of 1.0 nm; van der Waals interactions were likewise truncated at 1.0 nm using a smooth switching function (starting at 0.8 nm). All bonds involving hydrogen atoms were constrained using the LINCS algorithm (order 4, accuracy 1 × 10^−8^), enabling a stable integration time step of 2 fs. Before production MD, the system underwent a three-phase energy minimization protocol: (i) 3000 steps of steepest descent with position restraints applied to all non-hydrogen solute atoms (to relax solvent positions while preserving structural integrity); (ii) 2000 steps of conjugate gradient minimization with restraints on counterions only; and (iii) unconstrained minimization until the maximum force fell below 1000 kJ·mol^−1^·nm^−1^. Subsequent equilibration comprised: (a) 100 ps NVT heating from 0 K to 310 K using the Nosé–Hoover thermostat (coupling time constant τ_T_ = 0.5 ps); (b) an additional 100 ps NVT simulation at 310 K for temperature stabilization; and (c) 200 ps NPT equilibration at 310 K and 1 bar using the Parrinello–Rahman barostat (τ_P_ = 2.0 ps; compressibility 4.5 × 10^−5^ bar^−1^). Production molecular dynamics simulations were then performed in the NPT ensemble for 500 ns, with coordinates and energies saved every 10 ps. Trajectory analyses were conducted using standard GROMACS utilities: gmx rms for root-mean-square deviation (RMSD) relative to the initial minimized structure; gmx rmsf for per-residue root-mean-square fluctuation (RMSF); gmx hbond for hydrogen bond occupancy, lifetime, and donor–acceptor distance statistics; gmx gyrate for radius of gyration (Rg); gmx sasa for solvent-accessible surface area (SASA); and gmx sham for free energy landscape reconstruction from selected collective variables.

Binding free energies were estimated using the gmx_MMPBSA package (v1.5.0), interfaced with GROMACS 2022, for the MTOR–compound **11** complex. 500 evenly spaced frames were extracted from the final 50 ns of the production trajectory (i.e., every 100 ps), ensuring adequate sampling and statistical robustness. The internal (solute) dielectric constant was set to ε_in_ = 1, and the external (solvent) dielectric constant to ε_out_ = 80. Nonpolar solvation contributions were computed using the linear combination of pairwise overlaps (LCPO) method, with a surface tension coefficient of γ = 0.00542 kcal·mol^−1^·Å^−2^ and an offset parameter β = 0.

## 4. Conclusions

In this work, we systematically investigated four novel spiro-polycyclic sesquiterpene [4+2] trimers (Inubritantrimers A–D) and eight related synthetic intermediates from *Inula britannica* L. using an integrated in silico strategy to decipher the regulatory role of *exo*/*endo* stereoconfiguration on the structural properties, druggability, and anti-breast cancer activity of this rare class of natural products.

Quantum chemical calculations at the ωB97xD/6-311++G(2d,p) level revealed that *endo*-type cycloaddition products have superior intrinsic structural stability, while *exo*-type isomers exhibit enhanced electrophilic reactivity. All cycloaddition reactions were thermodynamically spontaneous, and the theoretically calculated spectral data were in excellent agreement with experimental results, validating the reliability of our computational workflow. ADMET screening identified compounds **5**–**9** and **11** as drug-like candidates, with acetoxy substitution found to be associated with increased toxicity risk.

Network pharmacology analysis demonstrated that these sesquiterpene trimers exert anti-breast cancer effects by modulating multiple targets in core oncogenic pathways, particularly the PI3K-Akt axis. Molecular docking results confirmed that all compounds showed robust binding affinity to seven key breast cancer targets, with compound **11** displaying the most potent and balanced binding activity, especially to MTOR (binding free energy −13.45 kcal/mol). 500 ns all-atom MD simulations and MM/PBSA calculations further validated the stable complex formation between compound **11** and MTOR (total binding free energy of −21.13 kcal/mol), with van der Waals interactions as the dominant driving force.

Collectively, our study, for the first time, constructed a complete stereochemistry-electronic structure–druggability–antitumor activity relationship for this rare class of sesquiterpene [4+2] trimers, and identified compound **11** as a novel MTOR-targeted lead compound with robust binding affinity and favorable druggability against triple-negative breast cancer. These findings not only provide atomic-level theoretical insights into the anti-breast cancer mechanism of sesquiterpene trimers from *Inula britannica* L., but also offer precise theoretical guidance and a rational design framework for the development of high-efficiency, low-toxicity targeted anti-breast cancer agents derived from natural sesquiterpene oligomers.

## Figures and Tables

**Table 1:** The thermodynamic properties for twelve compounds calculated in vacuum using the ωB97xD/6-311++G(2d,p) method.

Compd.	ZPE	G (kcal/mol)	H (kcal/mol)	S (J/mol/K)	U (kcal/mol)	ΔG (kcal/mol)
**1**	198.053	−507,665.970	−507,629.450	512.485	208.187	
**2**	183.104	−506,906.343	−506,869.253	520.481	193.404	
**3**	236.106	−651,387.826	−651,342.544	635.452	250.197	
**4**	205.717	−602,674.700	−602,633.258	581.572	218.040	
**5**	385.806	−1,014,588.385	−1,014,533.172	774.808	405.565	−16.07
**6**	407.647	−1,110,368.192	−1,110,308.492	837.788	429.462	−27.52
**7**	385.295	−1,014,593.906	−1,014,538.032	784.086	405.289	−21.59
**8**	423.067	−1,158,315.060	−1,158,250.332	908.332	447.120	−20.89
**9**	574.116	−1,521,507.815	−1,521,435.078	1020.725	603.477	−13.09
**10**	595.896	−1,617,297.803	−1,617,219.457	1099.451	627.685	−23.27
**11**	573.642	−1,521,521.382	−1,521,448.041	1029.205	603.097	−21.13
**12**	610.335	−1,665,231.371	−1,665,147.728	1173.779	644.412	−9.97

**Table 2:** UV–Vis absorption data for twelve compounds in methanol with oscillator strengths (*f*) above 0.2000 at the ωB97xD/6-311++G(2d,p) level of theory.

Compd.	Excited State	*E* (eV)	λ (nm)	*f*	Configuration (%)
**1**	3	5.9691	207.71	0.3159	*H − 2 → L (65.1)
	6	6.7468	183.77	0.4289	H → L + 2 (19.8)
**2**	3	5.9782	207.39	0.3143	H − 2 → L (66.0)
	6	6.7472	183.76	0.4352	H → L + 2 (22.8)
**3**	3	5.9792	207.36	0.3013	H − 1 → L (86.6)
	5	6.5878	188.20	0.2446	H → L + 7 (53.5)
**4**	6	6.0002	206.63	0.3212	H − 1 → L (84.3)
	17	7.1618	173.12	0.4029	H − 2 → L + 1 (22.6)
**5**	4	5.9876	207.07	0.2854	H − 4 → L (88.3)
	6	6.3454	195.39	0.2137	H − 1 → L + 1 (40.4)
	9	6.6724	185.82	0.2265	H → L + 1 (25.0)
**6**	4	6.0376	205.35	0.2535	H − 3 → L (77.5)
	7	6.3452	195.40	0.3501	H → L + 7 (27.4)
	11	6.6589	186.19	0.2999	H − 1 → L + 10 (29.5)
**7**	5	6.3047	196.65	0.2428	H → L + 9 (36.4)
	10	6.6583	186.21	0.3240	H − 1 → L + 9 (20.2)
**8**	7	6.3776	194.40	0.2140	H → L + 13 (39.0)
	9	6.6081	187.62	0.3162	H − 1 → L + 10 (28.0)
**9**	7	6.1972	200.06	0.2504	H − 6 → L (44.0)
	10	6.4191	193.15	0.2129	H → L + 26 (32.8)
	12	6.6208	187.27	0.2614	H → L + 1 (50.3)
**10**	5	5.9565	208.15	0.2306	H − 4 → L (69.2)
	9	6.4559	192.05	0.3804	H → L + 16 (16.6)
	14	6.6610	186.13	0.2985	H − 2 → L + 11 (30.0)
**11**	5	5.9420	208.66	0.2260	H − 5 → L (91.4)
	7	6.2756	197.57	0.2735	H → L + 10 (21.6)
	13	6.6366	186.82	0.2356	H − 1 → L (45.6)
**12**	5	5.9720	207.61	0.3599	H − 5 → L (62.9)
	10	6.3168	196.28	0.3180	H → L + 24 (56.5)
	14	6.6685	185.93	0.4001	H − 2 → L + 13 (25.7)

*H: highest occupied molecular orbital (HOMO); L: lowest unoccupied molecular orbital (LUMO).

**Table 3:** The frontier orbital energies and energy gaps for twelve compounds under a vacuum environment computed with ωB97XD/6-311++G(2d,p).

Compd.	E_HOMO_ (eV)	E_LUMO_ (eV)	∆*ε* (eV)
**1**	−9.01	0.47	9.47
**2**	−8.10	0.29	8.39
**3**	−9.13	0.39	9.52
**4**	−8.35	0.25	8.60
**5**	−8.80	0.28	9.08
**6**	−8.58	0.48	9.06
**7**	−8.74	0.48	9.22
**8**	−8.77	0.46	9.23
**9**	−8.34	0.17	8.51
**10**	−8.51	0.50	9.01
**11**	−8.49	0.53	9.02
**12**	−8.36	0.67	9.03

**Table 4:** Global reactivity indices and dipole moments for twelve compounds in vacuum (Vac), water (Wat), and methanol (Met) using the ωB97XD/6-311++G(2d,p) method.

Compd.	Env.	IP (eV)	EA (eV)	Χ (eV)	η (eV)	S (eV^−1^)	ω (eV)	Dipole Moment (Debye)
**1**	Vac	8.93	−0.27	4.33	4.60	0.22	2.03	6.85
	Wat	8.88	−0.28	4.30	4.58	0.22	2.02	8.65
	Met	8.88	−0.29	4.30	4.58	0.22	2.01	8.60
**2**	Vac	8.10	−0.29	3.90	4.20	0.24	1.81	4.91
	Wat	7.87	−0.26	3.80	4.07	0.25	1.78	6.24
	Met	7.88	−0.27	3.81	4.07	0.25	1.78	6.21
**3**	Vac	9.00	−0.18	4.41	4.59	0.22	2.12	6.13
	Wat	9.00	−0.25	4.38	4.63	0.22	2.07	8.34
	Met	9.01	−0.25	4.38	4.63	0.22	2.07	8.30
**4**	Vac	8.23	−0.11	4.06	4.17	0.24	1.98	8.59
	Wat	8.07	−0.25	3.91	4.16	0.24	1.83	10.97
	Met	8.07	−0.25	3.91	4.16	0.24	1.84	10.89
**5**	Vac	8.74	−0.09	4.32	4.42	0.23	2.11	7.66
	Wat	8.53	−0.28	4.12	4.40	0.23	1.93	11.21
	Met	8.53	−0.28	4.13	4.40	0.23	1.93	11.14
**6**	Vac	8.58	−0.48	4.05	4.53	0.22	1.81	6.99
	Wat	8.43	−0.27	4.08	4.35	0.23	1.91	9.94
	Met	8.44	−0.28	4.08	4.36	0.23	1.91	9.88
**7**	Vac	8.60	−0.25	4.17	4.42	0.23	1.97	6.71
	Wat	8.50	−0.28	4.11	4.39	0.23	1.93	9.76
	Met	8.51	−0.28	4.11	4.39	0.23	1.93	9.69
**8**	Vac	8.62	−0.24	4.19	4.43	0.23	1.99	5.99
	Wat	8.53	−0.26	4.14	4.40	0.23	1.95	8.97
	Met	8.54	−0.27	4.13	4.41	0.23	1.94	9.52
**9**	Vac	8.41	−0.19	4.11	4.30	0.23	1.97	6.45
	Wat	8.24	−0.30	3.97	4.27	0.23	1.85	4.75
	Met	8.27	−0.27	4.00	4.27	0.23	1.87	5.13
**10**	Vac	8.20	−0.24	3.98	4.22	0.24	1.88	14.17
	Wat	8.38	−0.31	4.03	4.35	0.23	1.87	17.01
	Met	8.39	−0.32	4.03	4.35	0.23	1.87	17.01
**11**	Vac	8.28	−0.41	3.93	4.35	0.23	1.78	12.94
	Wat	8.30	−0.33	3.98	4.32	0.23	1.84	14.48
	Met	8.29	−0.36	3.96	4.33	0.23	1.81	13.96
**12**	Vac	8.19	−0.44	3.87	4.31	0.23	1.74	7.91
	Wat	8.24	−0.33	3.96	4.28	0.23	1.83	11.79
	Met	8.24	−0.33	3.95	4.29	0.23	1.82	11.71

**Table 5:** Local reactivity indices and Mulliken charges for twelve compounds in vacuum using the ωB97XD/6-311++G(2d,p) method.

Compd.	Atom	*f*^+^ (eV)	*f^−^* (eV)	*f*^0^ (eV)	*s*^−^ (eV)	*s*^+^ (eV)	*ω*^−^ (eV)	*ω*^+^ (eV)	Mulliken (a.u.)
**1**	C3	4.417	15.520	9.968	0.960	3.373	8.988	31.582	0.395
	C6	7.084	26.891	16.987	1.539	5.844	14.416	54.722	−0.501
**2**	C1	3.879	0.593	2.236	0.924	0.141	7.038	1.077	−0.556
	C11	4.977	33.820	19.398	1.186	8.058	9.030	61.366	0.665
	H34	1.854	9.684	5.769	0.442	2.307	3.364	17.572	0.198
**3**	C3	4.889	14.710	9.799	1.065	3.204	10.345	31.127	0.350
	C6	6.036	20.690	13.363	1.315	4.507	12.772	43.783	−0.556
**4**	C1	3.767	0.499	2.133	0.903	0.120	7.449	0.986	−0.601
	C11	5.145	36.583	20.864	1.234	8.770	10.175	72.340	0.653
	C16	2.793	20.300	11.546	0.669	4.867	5.522	40.141	−0.478
**5**	C26	12.077	67.581	39.829	2.735	15.306	25.534	142.883	0.630
	H74	4.398	22.278	13.338	0.996	5.046	9.299	47.102	0.205
**6**	C12	17.095	3.428	10.262	3.773	0.757	30.920	6.200	−0.218
	C22	4.254	−0.341	1.957	0.939	−0.075	7.694	−0.617	0.160
	C26	1.229	31.908	16.569	0.271	7.042	2.223	57.711	0.682
	H76	1.764	23.927	12.846	0.389	5.281	3.190	43.276	0.203
**7**	C12	17.975	3.536	10.756	4.064	0.799	35.381	6.960	−0.399
	C22	3.972	−0.373	1.800	0.898	−0.084	7.819	−0.733	0.158
	C27	1.008	32.509	16.758	0.228	7.349	1.983	63.987	0.651
	H73	1.662	25.222	13.442	0.376	5.702	3.271	49.643	0.204
**8**	C9	17.078	3.553	10.316	3.857	0.802	33.905	7.053	−0.428
	C19	3.977	−0.369	1.804	0.898	−0.083	7.895	−0.733	−0.160
	C23	1.060	35.194	18.127	0.239	7.948	2.103	69.869	0.648
	H79	1.674	26.502	14.088	0.378	5.985	3.324	52.613	0.204
**9**	C41	22.083	11.858	16.970	5.139	2.760	43.395	23.301	0.064
	H103	7.649	8.312	7.980	1.780	1.934	15.030	16.333	0.199
**10**	C43	−36.492	26.895	−4.799	−8.651	6.376	−68.428	50.431	−0.492
	H114	0.140	10.975	5.558	0.033	2.602	0.263	20.580	0.186
	H97	14.090	−9.596	2.247	3.340	−2.275	26.421	−17.993	0.208
	H98	13.766	−10.070	1.848	3.263	−2.387	25.813	−18.882	0.215
**11**	C26	8.519	−10.647	−1.064	1.960	−2.450	15.151	−18.935	0.627
	C43	38.426	−37.980	0.223	8.841	−8.739	68.339	−67.544	−0.429
	H94	−12.641	13.561	0.460	−2.909	3.120	−22.481	24.116	0.208
	H95	−14.802	14.893	0.045	−3.406	3.427	−26.324	26.486	0.222
**12**	C40	5.435	43.115	24.275	1.260	9.993	9.457	75.014	−0.402
	C39	3.958	33.273	18.615	0.917	7.712	6.886	57.889	0.807

**Table 6:** Predicted ADMET and physicochemical properties of twelve compounds using the ACD/Percepta and ADMETlab 3.0 platforms.

Compd.	Phychem								Medchem		
	MW		LogS		TPSA		LogP		Lipinski		Reactive
	*a	*b	a	b	a	b	a	b	a	b	b
**1**	248.320	248.140	−3.010	−3.506	46.530	46.530	2.710	2.409	0	0	0.720
**2**	246.300	246.130	−3.310	−1.907	46.530	46.530	1.840	1.403	0	0	0.628
**3**	308.370	308.160	−4.310	−2.492	72.830	72.830	2.090	2.060	0	0	0.615
**4**	288.340	288.140	−3.750	−3.027	52.600	52.600	2.660	2.151	0	0	0.658
**5**	494.620	494.270	−3.410	−4.136	93.060	93.060	3.500	2.685	0	0	0.256
**6**	536.660	536.280	−3.860	−3.970	99.130	99.130	4.180	2.525	1	0	0.269
**7**	494.620	494.270	−3.360	−3.780	93.060	93.060	3.550	2.364	0	0	0.264
**8**	554.670	554.290	−3.880	−3.785	119.360	119.360	3.230	2.814	1	0	0.136
**9**	726.890	740.390	−2.660	−4.467	139.590	139.590	3.800	2.925	1	0	0.259
**10**	782.960	782.400	−3.580	−4.310	145.660	145.660	5.100	2.693	2	0	0.242
**11**	740.920	740.390	−3.140	−4.242	139.590	139.590	4.500	2.486	1	0	0.247
**12**	800.970	800.410	−3.580	−4.097	165.890	165.890	4.250	2.754	2	1	0.123
**Compd.**	**Metabolism**										
	**CYP1A2** **Inhibitor**		**CYP2C9** **Inhibitor**		**CYP2C19** **Inhibitor**		**CYP2D6** **Inhibitor**		**CYP3A4** **Inhibitor**		**HLM**
	**a**	**b**	**a**	**b**	**a**	**b**	**a**	**b**	**a**	**b**	**a**
**1**	0.370	0.003	0.380	0.050	0.460	0.008	0.420	0.003	0.620	0.015	0.785
**2**	0.400	0.002	0.350	0.000	0.420	0.024	0.370	0.000	0.390	0.027	0.197
**3**	0.410	0.001	0.390	0.002	0.490	0.001	0.360	0.002	0.610	0.191	0.501
**4**	0.490	0.000	0.350	0.000	0.410	0.006	0.430	0.000	0.540	0.084	0.402
**5**	0.250	0.000	0.360	0.000	0.440	0.000	0.360	0.000	0.550	0.015	0.725
**6**	0.270	0.000	0.360	0.000	0.460	0.000	0.410	0.000	0.580	0.008	0.602
**7**	0.250	0.000	0.350	0.001	0.450	0.001	0.360	0.000	0.530	0.015	0.052
**8**	0.280	0.000	0.380	0.000	0.460	0.000	0.380	0.000	0.540	0.345	0.273
**9**	0.220	0.000	0.370	0.000	0.430	0.000	0.360	0.000	0.560	0.090	0.979
**10**	0.220	0.000	0.370	0.000	0.430	0.000	0.350	0.000	0.580	0.000	0.747
**11**	0.220	0.000	0.370	0.000	0.430	0.000	0.360	0.000	0.570	0.007	0.583
**12**	0.240	0.000	0.380	0.000	0.430	0.000	0.360	0.000	0.570	0.049	0.311
**Compd.**	**Absorption**							**Distribution**			
	**Caco-2**		**HIA**		**P-gp Substrates**		**HOB**	**PPB**		**BBB**	**CNS**
	**a**	**b**	**a**	**b**	**a**	**b**	**b**	**a**	**b**	**b**	**a**
**1**	221.900	−4.581	100.000	0.006	0.420	0.109	0.979	84.000	91.228	0.056	−2.090
**2**	179.800	−5.052	100.000	0.023	0.420	0.578	0.965	83.000	57.113	0.607	−2.350
**3**	192.100	−4.766	100.000	0.000	0.420	0.105	0.741	85.000	58.459	0.050	−2.450
**4**	236.800	−4.844	100.000	0.056	0.430	0.364	0.967	85.000	83.837	0.129	−2.100
**5**	210.100	−5.183	100.000	0.000	0.450	0.532	0.993	95.000	81.621	0.476	−2.710
**6**	230.300	−5.078	100.000	0.000	0.450	0.885	0.996	96.000	92.162	0.122	−2.640
**7**	210.900	−5.339	100.000	0.000	0.440	0.889	0.992	95.000	92.014	0.241	−2.720
**8**	202.000	−5.450	100.000	0.000	0.490	0.490	0.950	93.000	70.534	0.076	−2.830
**9**	166.500	−5.414	100.000	0.000	0.530	0.609	0.999	97.000	81.268	0.551	−3.240
**10**	124.300	−5.378	100.000	0.000	0.570	0.988	1.000	98.000	92.801	0.011	−3.160
**11**	134.300	−5.586	100.000	0.000	0.540	0.988	1.000	98.000	91.207	0.058	−3.190
**12**	151.100	−5.605	100.000	0.000	0.640	0.703	0.997	97.000	76.526	0.136	−3.240
**Compd.**	**Toxicity**								**Excretion**		
	**AMEs**		**hERG**		**DILI**	**ROA**	**Neurotoxicity-DI**		**T_1/2_**	**Cl**	
	**a**	**b**	**a**	**b**	**b**	**b**	**b**		**b**	**b**	
**1**	0.430	0.916	0.300	0.036	0.843	0.533	0.536		1.724	13.340	
**2**	0.430	0.678	0.340	0.084	0.941	0.443	0.796		2.077	8.114	
**3**	0.310	0.870	0.300	0.051	0.830	0.466	0.774		0.964	6.640	
**4**	0.420	0.794	0.320	0.065	0.979	0.477	0.827		1.523	8.305	
**5**	0.350	0.798	0.340	0.026	0.902	0.307	0.241		1.743	9.548	
**6**	0.360	0.899	0.340	0.013	0.971	0.237	0.215		1.540	9.449	
**7**	0.350	0.886	0.350	0.017	0.958	0.179	0.194		1.937	9.967	
**8**	0.370	0.807	0.360	0.025	0.947	0.137	0.414		1.504	5.469	
**9**	0.360	0.758	0.380	0.016	0.936	0.363	0.150		2.013	6.830	
**10**	0.370	0.919	0.380	0.010	0.961	0.426	0.118		1.990	7.926	
**11**	0.360	0.909	0.380	0.009	0.960	0.544	0.088		2.313	7.714	
**12**	0.380	0.885	0.400	0.013	0.964	0.443	0.209		1.782	3.803	

*a: ACD/Percepta; *b: ADMETLab 3.0.

**Table 7:** Comparison of molecular docking binding free energies (kcal/mol) between compound **11** and clinically specific inhibitors against core anticancer targets.

Target	MTOR	AKT1	BCL2
Compd.			
**Rapamycin**	−14.71		
**MK-2206**		−10.74	
**Venetoclax**			−10.31
**compound 11**	−13.45	−9.77	−10.53

**Table 8:** Statistics of interactions from molecular docking between compounds **6**, **9**, **11**, and seven core anti-breast cancer target proteins.

Compd.	Target	Hydrogen Bonding	Hydrophobic Interaction
**6**	EFGR	THR766	PHE699/VAL7919/LYS721/LEU820
	ESR1	MET342/ASN407/LEU409/ASN413	LEU408/VAL418
	MDM2	TYR100	LYS51/LEU54/PHE55/ILE61/TYR67/VAL93/ILE99/TYR100
	TNF	GLY121/GLN125	LEU55/TYR119/TYR151
	BCL2	THR91/PHE112	PHE63/TYR67/ASP70/PHE71/PRO82/PHE83/VAL92/GLU95/PHE112
	MTOR	TYR57/TYR113	TYR57/VAL86/ILE87/TRP90/TYR113/PHE130/PHE2039
	AKT1	ASN53/TRP80	ASN53/GLN79/TRP80/LEU213/LYS268/VAL270
**9**	EFGR	GLU738/LYS851	ALA698/PHE699/LEU723/GLU734/ILE735/LEU834/LYS851
	ESR1	ASP332/ASN407/LEU409	ASP332/LEU345/ASN407
	MDM2	LYS94/HIS96	LYS51/LEU54/PHE55/VAL93
	TNF	SER60/LEU120/GLY121/TYR151	LEU55/TYR59/TYR119/TYR119
	BCL2	TYR67/LEU96	TYR67/ASP70/MET74/PRO82/PHE83/VAL92/GLU95/LEU96
	MTOR	ASP68/GLN85/LYS121	VAL86/ILE87/TYR113/LYS121/PHE2039
	AKT1	ASP274/ASN279/PHE293/LYS297	VAL164/PHE293/LYS297
**11**	EFGR	ARG817/LYS851	ALA698/PHE699/VAL702/GLU734/ARG817/LEU834/LYS851
	ESR1	ASN519	GLU380/THR460/LEU525/TYR526
	MDM2	GLN24/LEU54/GLY58	LEU54/PHE55/ILE61/PHE91/VAL93/ILE99
	TNF	GLY121/TYR151	LEU55/LEU57/TYR59/TYR119
	BCL2	GLU73	PHE93/TYR67/GLU73/MET74/PRO82/THR91/VAL92/GLU95/LEU96/ARG105
	MTOR	TYR2038	PHE67/PHE77/GLN85/VAL86/TYR113/LYS121/PHE130/TYR2038/PHE2039/TYR2105
	AKT1	LYS154/LYS158/GLU278/THR291	GLU234/PHE236/PHE237/GLU278/PHE293/LEU295

## Data Availability

All data generated or analyzed during this study are included in this published article and its Supplementary Information Files.

## Supplementary Materials

The following supporting information can be downloaded at https://www.mdpi.com/article/10.3390/molecules31101759/s1: Table S1. Theoretical and experimental values of main bond lengths (Å), bond angles (°), and dihedral angles (°) of twelve compounds optimized in vacuum (Vac), water (Wat), and methanol (Met) environments. Table S2. Theoretical and experimental ^13^C and ^1^H NMR chemical shift values of eight compounds using TMS as the standard for C and H at the ωB97XD/6-311++G(2d,p) level. Table S3. Theoretical and experimental values (cm^−1^) of the IR vibrational frequencies of compounds **6**, **7**, **9**–**12** in vacuum, calculated using ωB97XD/6-311++G(2d,p). Table S4. Relevant values and proportions of molecular surface analysis for twelve compounds. Figure S1. UV-Vis absorption spectra of twelve compounds in methanol calculated using ωB97XD/6-311++G(2d,p) method. Figure S2. Frontline molecular orbital diagrams of twelve compounds in a vacuum environment. Figure S3. Surface area distribution diagram of the electrostatic potential interval of the compounds in vacuum. Figure S4. Molecular docking diagrams of compounds **5**–**9** and **11** with targets EGFR, MDM2, EXR1, TNF, BCL2, MTOR, and AKT1.
