# Supplementary material for: A Computational Investigation of Four Sesquiterpene [4+2] Trimers, Inubritantrimers A–D, and Their Synthetic Intermediates Isolated from Inula britannica L
Source: Molecules. 2026 May 20;31(10):1759. doi: 10.3390/molecules31101759 (PMC13209964; doi:10.3390/molecules31101759)
Supplement: Supplementary file 1 [file molecules-31-01759-s001.zip › molecules-4264880-supplementary.pdf]

Supplementary Information

A computational investigation of four sesquiterpene [4+2] trimers, Inubritantrimers A–D, and their synthetic intermediates isolated from *Inula britannica* L.

Xiaoyun Xia [1], Xiandong Du [1], Zhifeng Chen [1], Sisi Yu [1] and Chaojie Wang [1,*]

[1] School of Pharmaceutical Sciences, Wenzhou Medical University, Wenzhou 325035, China;
* Correspondence: chjwang@wmu.edu.cn; Tel.: +86135-6628-6518

Table S1. Theoretical and experimental values of main bond lengths (Å), bond angles (°), and dihedral angles (°) of twelve compounds optimized in vacuum (Vac), water (Wat), and methanol (Met) environments.

Compd.	Bond length	Vac	Wat	Met	Exp.	Compd.	Bond angle	Vac	Wat	Met	Exp.
**1**	C12-O17	1.418	1.422	1.422		**1**	O5-C1-C8	109.238	108.792	108.792	
	C3=C6	1.321	1.322	1.322		**2**	O13-C8-C9	109.469	109.526	109.142	
**2**	C1-C2	1.333	1.333	1.333		**3**	O5-C1-C8	109.477	109.161	109.454	
	C5-O18	1.420	1.424	1.423		**4**	O13-C8-C9	109.368	109.410	109.407	
	C11=C16	1.322	1.323	1.323		**5**	O13-C9-C10	110.407	110.434	110.435	108.400
**3**	C9-O22	1.434	1.438	1.438			C14-C24-O25	119.311	118.969	118.978	118.830
	C10-C13	1.522	1.522	1.522			C11-C15-C16	103.894	103.885	103.885	103.400
	C3=C6	1.322	1.323	1.323			C14-C24-C16	93.700	93.951	93.945	94.800
**4**	C4-C5	1.430	1.440	1.440		**6**	O13-C9-C10	110.419	110.446	110.446	110.540
	C11=C16	1.322	1.323	1.323			C14-C23-O32	113.415	112.958	112.969	115.500
**5**	C9-O13	1.437	1.449	1.448	1.460		C11-C39-C16	104.991	105.027	105.026	104.700
	C1-O30	1.418	1.423	1.423	1.423		C14-C23-C16	94.889	94.913	94.913	94.500
	C11-C15	1.563	1.565	1.565	1.569	**7**	O13-C9-C10	110.405	110.452	110.448	
	C11-C14	1.601	1.602	1.602	1.611		C14-C23-O33	114.545	114.164	114.131	
	C24-O25	1.390	1.401	1.401	1.410		C11-C24-C16	105.035	105.070	105.060	
	C14-C24	1.558	1.555	1.555	1.572		C14-C23-C16	94.037	94.131	94.135	
	C24-C16	1.542	1.539	1.539	1.531	**8**	O10-C6-C7	110.181	110.298	110.297	
**6**	C6-O29	1.417	1.423	1.423	1.436		C4-C2-C1	120.925	120.833	120.836	
	C9-O13	1.441	1.450	1.450	1.448		C11-C20-C13	94.137	94.137	94.136	
	C11-C39	1.543	1.544	1.544	1.545		C8-C40-C13	105.032	105.072	105.071	
	C39-C16	1.566	1.566	1.566	1.571		C11-C20-O28	114.515	114.155	114.163	
	C23-O32	1.424	1.424	1.424	1.423	**9**	013-C9-C10	110.393	110.293	110.295	
	C14-C23	1.528	1.529	1.529	1.511		C11-C15-C16	103.920	103.997	103.996	
	C23-C16	1.538	1.538	1.538	1.539		C14-C24-O25	113.266	112.433	112.435	
**7**	C9-O13	1.440	1.450	1.450			C14-C24-C16	94.569	94.649	94.648	
	C6-O30	1.417	1.423	1.422			C26-C38-C32	106.159	105.714	105.709	
	C11-C14	1.571	1.572	1.572			C38-C39-C30	94.047	94.245	94.243	
	C24-C16	1.561	1.562	1.562			C11-C14-C16	75.713	75.800	75.800	
	C23-O33	1.400	1.404	1.404		**10**	O13-C9-C8	109.634	110.366	110.366	109.911
	C14-C23	1.535	1.536	1.535			C14-C23-C16	94.502	94.710	94.710	95.250
	C23-C16	1.553	1.552	1.552			C14-C23-O53	113.586	116.680	116.692	113.930
**8**	O10-C6	1.441	1.450	1.450			C11-C16-C14	38.710	38.671	38.672	38.830
	C3-O38	1.431	1.435	1.435			C27-C33-C32	103.933	103.860	103.859	103.280
	C8-C40	1.543	1.544	1.544		**11**	013-C9-C10	110.170	110.307	110.304	110.000
	C40-C13	1.560	1.561	1.561			C14-C23-C16	93.977	94.017	94.014	94.050
	C11-C20	1.536	1.536	1.536			C14-C23-O53	113.341	114.188	114.198	116.240
	C20-C13	1.553	1.552	1.552			C11-C16-C14	38.758	38.487	38.587	38.720
**9**	C9-O13	1.431	1.439	1.439			C20-C19-O25	105.101	104.907	104.916	105.290
	C11-C15	1.557	1.558	1.558			C27-C28-C29	103.679	103.110	103.108	104.030
	C15-C16	1.537	1.538	1.538			C28-C29-C30	102.933	101.540	101.539	103.400
	C16-C24	1.538	1.534	1.535			C27-C33-C32	103.851	104.546	104.554	103.351
	C14-C24	1.546	1.545	1.545							
	C24-O25	1.406	1.408	1.408		**12**	O10-C6-C7	109.943	110.228	110.222	
	C20-O28	1.436	1.445	1.444			C11-C20-O47	114.639	114.247	114.255	
	C26-C29	1.552	1.555	1.555			C8-C13-C11	38.041	38.437	38.446	
	C29-C30	1.537	1.535	1.536			C24-C25-C26	103.610	103.693	103.692	
	C38-C39	1.567	1.567	1.567			C25-C26-C27	101.062	101.408	101.402	

	C39-C30	1.531	1.528	1.528	
**10**	C23-O53	1.428	1.428	1.428	1.442
	C11-C24	1.541	1.543	1.543	1.551
	C16-C24	1.563	1.563	1.563	1.564
	C20-H74	1.096	1.095	1.095	1.095
	C19-O25	1.436	1.447	1.446	1.465
	C27-C28	1.564	1.565	1.565	1.556
	C28-C29	1.546	1.547	1.547	1.548
	C29-C30	1.545	1.541	1.541	1.522
	C30-C33	1.560	1.558	1.558	1.554
	C34-C40	1.530	1.529	1.529	1.534
**11**	C9-O13	1.444	1.450	1.450	1.459
	C23-O51	1.410	1.404	1.404	1.416
	C11-C24	1.540	1.543	1.543	1.542
	C24-C16	1.562	1.562	1.562	1.571
	H71-C20	1.095	1.094	1.094	0.980
	C19-O25	1.436	1.443	1.443	1.455
	C27-C28	1.564	1.567	1.567	1.564
	C28-C29	1.546	1.545	1.545	1.549
	C29-C30	1.545	1.528	1.528	1.537
	C30-C33	1.560	1.556	1.557	1.564
	C34-C40	1.530	1.529	1.529	1.528
**12**	C1-C2	1.520	1.521	1.521	
	C6-C10	1.442	1.449	1.449	
	C20-O47	1.400	1.405	1.405	
	C8-C21	1.542	1.543	1.543	
	C21-C13	1.562	1.563	1.563	
	C24-C25	1.570	1.572	1.572	
	C25-C26	1.544	1.543	1.543	
	C26-C27	1.526	1.526	1.526	
	C27-C30	1.562	1.563	1.563	
**MAD**		0.012	0.010	0.010	

	C24-C30-C29	104.799	106.276	106.280	

**Compd.**	**Dihedral angle**	**Vac**	**Wat**	**Met**	**Exp.**
**5**	C11-C15-C16-C23	-66.222	-66.357	-66.354	69.790
	C11-C15-C16-C24	38.285	38.063	38.068	41.120
**6**	C39-C11-C14-C15	-66.315	-65.852	-65.865	67.100
	C16-C23-O32-C34	170.247	170.281	170.347	171.271
**7**	C24-C11-C14-C15	-67.264	-66.448	-66.352	
	C11-C24-C16-C23	-36.775	-35.996	-35.894	
	C16-C23-O33-H66	-53.279	-51.999	-51.965	
**8**	C8-C40-C13-C19	65.531	66.307	66.292	
	C8-C40-C13-C20	-37.019	-36.189	-36.205	
	C13-C20-O28-H62	-53.128	-52.048	-52.059	
**9**	C15-C11-C14-C24	36.711	37.283	37.275	
	C15-C11-C14-C17	-63.456	-63.100	-63.111	
	C16-C24-C14-C11	-56.228	-56.627	-56.625	
	C26-C29-C30-C39	42.635	43.127	43.117	
	C26-C29-C30-C31	-62.344	-61.778	-61.790	
**10**	C27-C28-C29-C30	36.943	36.569	36.581	38.135
	C27-C33-C32-C31	-70.625	-70.700	-70.696	-70.705
**11**	C22-C11-C14-C15	-66.118	-65.202	-65.216	66.300
	C14-C23-C16-C24	56.799	58.209	58.215	58.030
	H71-C20-C19-O25	-82.494	-83.118	-83.099	84.460
	C28-C29-C30-C33	-56.281	-58.718	-58.720	55.100
	C27-C28-C29-C30	37.079	35.026	35.025	35.900
	C27-C33-C32-C31	-71.109	-71.186	-71.185	70.710
**12**	C8-C21-C13-C20	-34.267	-33.621	-33.629	
	C21-C8-C11-C12	-64.647	-63.969	-63.978	
	C24-C25-C26-C27	-41.561	-40.934	-40.944	
	C25-C26-C27-C30	58.503	58.087	58.092	
	C24-C30-C29-C28	67.429	67.367	67.365	
**MAD**		0.892	1.098	1.093	

Table S2. Theoretical and experimental ^{13}C and ^{1}H NMR chemical shift values of eight compounds using TMS as the standard for C and H at the ωB97XD/6-311++G(2d,p) level.

5	Theo.	Exp.	6	Theo.	Exp.	7	Theo.	Exp.	8	Theo.	Exp.
1-C	76.9	75.3	1-C	27.0	26.0	1-C	27.0	26.2	1-C	35.5	32.2
2-C	28.4	26.4	2-C	32.3	29.7	2-C	31.8	29.9	2-C	163.8	150.3
3-C	30.3	30.4	3-C	43.2	38.2	3-C	42.7	38.2	3-C	72.5	69.7
4-C	34.8	38.8	4-C	160.2	149.5	4-C	160.1	148.3	4-C	125.6	117.3
5-C	160.4	150.9	5-C	43.1	38.5	5-C	43.3	38.5	5-C	47.2	41.7
6-C	43.0	41.1	6-C	84.5	80.7	6-C	84.2	81.0	6-C	77.8	74.8
7-C	126.1	118.1	7-C	125.8	118.1	7-C	125.9	119.2	7-C	44.3	41.4
8-C	41.7	44.0	8-C	47.2	42.2	8-C	47.9	41.7	8-C	57.1	57.6
9-C	81.1	76.7	9-C	78.1	75.3	9-C	78.8	75.5	9-C	187.1	179.4
10-C	41.3	37.3	10-C	42.5	38.5	10-C	42.2	38.5	11-C	64.5	57.6
11-C	64.0	58.1	11-C	57.1	56.8	11-C	56.9	57.1	12-C	144.3	137.0
12-C	196.0	185.6	12-C	186.8	178.8	12-C	187.0	179.7	13-C	66.4	63.4
14-C	72.3	70.0	14-C	61.0	56.2	14-C	65.3	57.9	14-C	38.2	29.5
15-C	43.5	35.3	15-C	142.8	134.2	15-C	143.8	134.0	15-C	39.7	36.1
16-C	57.1	51.9	16-C	64.3	62.3	16-C	65.2	63.4	16-C	85.5	82.8
17-C	144.1	140.0	17-C	38.2	29.8	17-C	38.4	29.6	17-C	48.4	45.2
18-C	30.5	24.9	18-C	39.8	36.1	18-C	39.9	36.2	18-C	28.9	26.4
19-C	44.1	45.4	19-C	85.5	82.5	19-C	85.2	82.8	19-C	143.9	133.8
20-C	81.3	81.5	20-C	48.7	45.3	20-C	48.8	45.4	20-C	93.5	82.6
21-C	42.4	37.3	21-C	28.7	26.0	21-C	28.7	26.3	22-C	177.2	170.2
22-C	33.1	26.3	22-C	144.1	136.4	22-C	143.8	136.9	23-C	149.0	139.7
23-C	151.7	134.4	23-C	93.8	81.9	23-C	93.0	82.7	26-C	21.6	23.4
24-C	89.2	85.5	25-C	177.4	170.0	24-C	40.5	35.7	27-C	28.2	29.1
26-C	151.8	139.2	26-C	148.9	139.4	26-C	177.8	170.2	29-C	18.4	17.1
27-C	176.8	170.2	30-C	25.0	23.0	27-C	148.4	139.7	30-C	16.5	14.3
29-C	19.8	21.4	31-C	24.8	21.6	31-C	24.8	22.9	31-C	38.8	34.5
31-C	20.8	21.6	34-C	179.6	170.0	32-C	24.9	21.6	32-C	31.0	26.6
32-C	21.1	18.9	35-C	23.7	21.2	34-C	18.2	17.1	33-C	67.4	64.3
35-C	14.4	13.7	36-C	19.1	17.0	35-C	16.0	14.3	36-C	179.7	171.1
36-C	126.9	119.5	37-C	16.1	14.3	36-C	126.1	119.4	37-C	23.4	21.0
37-H	3.70	3.30	38-C	126.6	119.5	37-H	1.83	1.82	39-C	126.7	119.3
38-H	1.52	1.80	39-C	40.8	36.4	38-H	1.54	1.61	40-C	41.1	35.5
42-H	2.29	2.43	40-H	1.87	1.82	39-H	1.58	1.60	41-H	2.63	2.42
43-H	5.10	4.63	41-H	1.55	1.62	40-H	1.50	1.54	42-H	5.75	5.36
44-H	3.37	3.31	42-H	1.57	1.65	41-H	2.41	2.43	43-H	2.75	3.33
45-H	5.07	4.79	43-H	1.52	1.57	42-H	3.29	3.24	44-H	4.97	4.88
46-H	1.31	1.41	44-H	2.35	2.45	43-H	5.97	5.40	45-H	2.53	2.54
47-H	2.12	2.32	45-H	3.38	3.28	44-H	2.79	3.27	46-H	1.57	1.88
48-H	1.98	2.03	46-H	5.78	5.37	45-H	5.03	4.90	47-H	2.72	2.69
49-H	2.16	2.28	47-H	2.96	2.79	46-H	2.36	2.57	48-H	2.14	2.25
50-H	2.43	2.58	48-H	5.03	4.89	47-H	1.50	1.52	49-H	2.16	2.36
51-H	2.75	2.16	49-H	2.39	2.59	48-H	2.69	2.67	50-H	1.96	2.01
52-H	2.51	3.00	50-H	1.49	1.53	49-H	2.14	2.23	51-H	4.28	4.20
53-H	3.10	2.71	51-H	2.99	2.86	50-H	2.14	2.37	52-H	2.69	2.84
54-H	4.34	4.26	52-H	2.09	2.15	51-H	2.10	2.01	53-H	2.13	2.04
55-H	2.44	2.29	53-H	2.17	2.36	52-H	4.24	4.19	54-H	3.20	3.00
56-H	1.65	2.02	54-H	2.00	2.00	53-H	2.74	2.80	55-H	3.69	3.64
57-H	2.57	2.67	55-H	4.31	4.21	54-H	2.13	2.06	57-H	0.84	1.10

58-H	3.67	3.42	56-H	2.70	2.82	55-H	3.26	3.00	58-H	0.87	1.10
62-H	1.30	1.09	57-H	2.16	2.08	56-H	3.72	3.62	61-H	1.37	1.32
65-H	0.96	1.15	58-H	3.20	3.03	57-H	1.83	1.77	63-H	0.91	1.07
67-H	1.16	1.15	59-H	4.45	4.60	58-H	1.88	1.96	64-H	0.91	1.07
68-H	0.92	1.15	62-H	0.99	1.11	60-H	0.88	1.09	68-H	1.64	1.63
70-H	1.56	1.72	64-H	1.10	1.19	61-H	0.94	1.09	69-H	1.07	1.39
71-H	1.89	1.72	65-H	0.90	1.19	63-H	1.07	1.18	72-H	1.22	1.47
72-H	1.58	1.72	67-H	2.14	2.08	64-H	0.96	1.18	73-H	3.48	3.96
73-H	5.91	5.58	68-H	2.12	2.08	67-H	0.86	1.06	74-H	4.51	4.06
74-H	6.52	6.23	70-H	0.95	1.05	68-H	0.95	1.06	76-H	2.14	2.00
			71-H	1.09	1.05	71-H	1.59	1.64	77-H	2.17	2.00
			75-H	1.54	1.67	72-H	1.58	1.64	80-H	5.91	6.20
			76-H	6.57	6.22	73-H	6.56	6.20	81-H	1.81	1.94
			77-H	5.92	5.54	74-H	5.82	5.52	82-H	1.80	1.94
			78-H	1.84	1.88						
			79-H	1.88	2.00						

**9**	**Theo.**	**Exp.**	**10**	**Theo.**	**Exp.**	**11**	**Theo.**	**Exp.**	**12**	**Theo.**	**Exp.**
1-C	82.9	79.0	1-C	26.8	25.9	1-C	26.8	26.1	1-C	35.9	32.2
2-C	26.4	26.0	2-C	32.3	29.7	2-C	32.2	29.8	2-C	162.2	150.7
3-C	31.5	29.9	3-C	43.1	38.1	3-C	42.7	38.2	3-C	72.5	69.7
4-C	41.0	38.1	4-C	160.6	149.6	4-C	159.0	148.2	4-C	125.6	117.0
5-C	157.4	150.0	5-C	43.0	37.2	5-C	43.0	41.6	5-C	47.7	41.7
6-C	42.7	38.6	6-C	84.6	80.5	6-C	84.8	80.9	6-C	77.0	74.5
7-C	134.4	119.4	7-C	125.2	117.7	7-C	126.4	119.0	7-C	44.3	41.3
8-C	40.6	40.0	8-C	45.7	42.2	8-C	44.4	41.6	8-C	56.5	56.2
9-C	81.5	77.5	9-C	77.9	74.8	9-C	78.2	75.0	9-C	186.7	178.8
10-C	45.5	39.5	10-C	42.5	39.6	10-C	42.1	39.9	11-C	65.0	57.2
11-C	60.8	59.9	11-C	60.2	55.9	11-C	61.3	56.3	12-C	142.8	133.3
12-C	190.9	185.6	12-C	188.2	178.1	12-C	189.0	178.9	13-C	64.8	62.7
14-C	73.5	68.2	14-C	58.7	55.6	14-C	60.0	57.3	14-C	38.2	29.6
15-C	41.4	35.8	15-C	142.8	133.5	15-C	143.1	133.4	15-C	40.3	36.5
16-C	56.1	53.0	16-C	63.9	61.3	16-C	66.7	62.5	16-C	81.6	83.8
17-C	147.4	141.6	17-C	34.0	29.6	17-C	33.8	38.5	17-C	51.2	44.5
18-C	32.6	24.3	18-C	38.9	38.5	18-C	39.1	36.6	18-C	27.7	26.6
19-C	55.1	46.1	19-C	86.5	83.5	19-C	86.7	83.9	19-C	147.2	137.8
20-C	82.8	79.6	20-C	46.5	44.5	20-C	46.9	44.4	20-C	94.1	36.5
21-C	40.5	37.0	21-C	29.6	26.8	21-C	29.7	27.1	21-C	41.8	36.4
22-C	29.8	25.9	22-C	147.6	137.2	22-C	147.5	137.6	23-C	188.3	187.0
23-C	145.6	136.4	23-C	86.2	82.2	23-C	87.1	83.3	24-C	63.1	57.4
24-C	87.9	85.4	24-C	41.1	36.4	24-C	39.7	36.6	25-C	34.9	32.8
26-C	62.9	58.1	26-C	196.2	170	26-C	196.0	187.1	26-C	55.3	53.5
27-C	191.7	169.3	27-C	62.4	57.4	27-C	62.0	57.3	27-C	90.9	84.8
29-C	32.9	32.3	28-C	36.3	32.8	28-C	36.5	32.8	28-C	143.6	138.5
30-C	56.9	52.0	29-C	57.5	53.4	29-C	57.4	53.5	29-C	148.4	141.8
31-C	151.0	134.0	30-C	88.6	84.7	30-C	88.7	84.7	30-C	72.2	67.3
32-C	142.2	138.8	31-C	148.5	137.2	31-C	147.6	138.5	31-C	36.4	28.8
33-C	27.8	25.6	32-C	146.2	138.7	32-C	146.9	136.9	32-C	40.8	39.2
34-C	49.7	45.1	33-C	70.6	67.2	33-C	70.9	67.2	33-C	81.9	79.1
35-C	84.8	81.2	34-C	33.1	28.8	34-C	33.0	28.9	34-C	43.1	40.9
36-C	37.9	37.0	35-C	42.3	39.1	35-C	42.1	39.2	35-C	34.4	26.6
37-C	34.5	29.9	36-C	81.7	79	36-C	81.8	79.0	36-C	14.2	12.7

38-C	74.3	68.2	37-C	44.6	40.7	37-C	44.6	40.8	37-C	22.7	19.2
39-C	92.3	85.8	38-C	29.8	26.5	38-C	30.0	26.5	39-C	151.3	141.8
40-C	152.2	140.1	39-C	13.8	14.4	39-C	14.1	12.7	40-C	126.8	119.7
41-C	178.0	169.3	40-C	21.6	19.3	40-C	21.5	19.3	41-C	177.7	170.5
43-C	21.9	21.2	42-C	151.8	141.6	42-C	151.9	141.6	45-C	21.2	23.2
45-C	25.6	22.9	43-C	126.5	119.8	43-C	126.6	120.0	46-C	31.6	29.4
46-C	17.9	19.1	44-C	177.8	170.5	44-C	177.7	170.6	48-C	18.1	17.1
47-C	19.8	18.1	49-C	24.8	23.1	49-C	25.0	23.1	50-C	16.5	14.5
48-C	125.5	118.4	50-C	25.0	21.7	50-C	24.8	21.6	51-C	38.9	34.2
53-C	14.4	12.9	51-C	18.1	17	51-C	18.3	17.1	52-C	31.1	27.0
54-C	16.0	14.3	52-C	17.1	14.4	52-C	17.1	14.6	53-C	67.5	64.4
58-H	1.67	1.60	55-C	179.3	170	55-H	1.85	1.84	56-C	179.4	170.9
60-H	2.56	2.38	56-C	23.7	21.2	56-H	1.54	1.61	57-C	23.4	21.1
61-H	6.10	4.69	58-H	1.85	1.83	58-H	1.48	1.24	59-H	2.62	2.44
62-H	3.60	2.64	59-H	1.54	1.64	59-H	2.34	2.45	60-H	5.81	5.35
63-H	5.19	4.87	60-H	1.56	1.60	61-H	5.82	5.40	61-H	2.74	3.38
64-H	2.26	2.28	61-H	1.55	1.54	62-H	3.45	3.34	62-H	4.94	4.84
65-H	1.42	1.54	62-H	2.38	2.45	63-H	4.96	4.86	63-H	2.32	2.53
66-H	2.49	2.27	63-H	3.33	3.26	64-H	2.27	2.56	64-H	1.92	1.88
67-H	1.16	1.94	64-H	5.83	5.35	65-H	1.38	1.53	65-H	2.69	2.68
69-H	2.53	2.27	65-H	2.62	2.75	66-H	2.67	2.64	66-H	2.10	2.23
70-H	1.70	1.71	66-H	4.91	4.82	67-H	2.00	2.20	67-H	2.16	1.97
71-H	2.18	2.74	67-H	2.33	2.56	68-H	2.27	1.95	68-H	1.91	2.41
72-H	4.16	4.33	68-H	1.42	1.51	69-H	1.97	1.75	69-H	4.33	4.26
73-H	1.95	1.94	69-H	2.96	2.80	70-H	4.32	4.27	70-H	2.53	3.18
74-H	2.23	2.38	70-H	2.13	2.14	71-H	2.30	2.24	71-H	1.99	2.33
75-H	2.62	2.90	71-H	2.30	2.37	72-H	1.73	1.82	73-H	3.72	3.58
76-H	3.65	3.59	72-H	1.95	2.09	73-H	2.37	2.30	74-H	1.79	1.83
78-H	1.74	1.90	73-H	4.36	4.26	74-H	3.65	3.57	75-H	1.97	2.18
79-H	1.77	2.27	74-H	2.28	2.23	75-H	1.96	2.40	76-H	1.47	1.83
80-H	2.35	2.59	75-H	1.82	1.85	76-H	1.89	1.98	77-H	2.13	2.68
81-H	3.36	2.83	76-H	2.38	1.85	77-H	1.71	1.83	79-H	3.68	3.57
82-H	2.31	2.19	77-H	4.31	4.49	78-H	2.01	2.16	80-H	2.47	2.64
83-H	3.33	2.51	78-H	2.08	2.38	79-H	2.49	2.64	81-H	2.33	2.47
84-H	4.06	4.24	79-H	2.02	1.96	80-H	3.62	3.56	82-H	1.64	1.82
85-H	2.30	2.38	80-H	1.76	1.82	81-H	2.44	2.63	83-H	4.53	4.26
86-H	1.85	1.94	81-H	2.03	2.14	82-H	2.41	2.46	84-H	3.07	3.18
87-H	2.58	2.29	82-H	2.49	2.80	83-H	1.58	1.82	85-H	2.99	2.84
88-H	4.24	3.45	83-H	3.60	3.56	84-H	4.29	4.26	86-H	2.77	2.33
90-H	1.18	1.10	84-H	2.41	2.55	85-H	3.31	3.22	87-H	1.64	1.70
91-H	1.06	1.10	85-H	2.42	2.45	86-H	2.97	2.87	90-H	1.33	1.19
93-H	1.22	1.13	86-H	1.59	1.83	87-H	2.36	2.34	93-H	6.50	6.15
97-H	1.06	1.19	87-H	4.34	4.26	88-H	1.58	1.70	94-H	5.83	5.49
98-H	1.11	1.19	88-H	3.31	3.19	90-H	1.56	1.70	97-H	1.02	1.10
101-H	0.74	1.23	89-H	2.94	2.32	93-H	1.05	1.21	100-H	1.31	1.32
102-H	6.45	6.17	90-H	2.36	1.83	94-H	6.46	6.16	103-H	1.07	1.06
103-H	5.87	5.43	91-H	1.66	1.70	95-H	5.81	5.52	108-H	1.52	1.56
106-H	1.72	1.74	93-H	1.58	1.70	98-H	1.01	1.16	109-H	1.08	1.44
109-H	1.99	1.85	96-H	1.06	1.20	100-H	1.03	1.18	111-H	1.94	1.54
			97-H	6.46	6.15	103-H	1.05	1.06	112-H	1.24	1.61
			98-H	5.79	5.48	104-H	1.05	1.06	113-H	3.54	4.04

	101-H	0.99	1.15	106-H	1.84	1.57	
	103-H	1.02	1.15	107-H	1.50	1.57	
	106-H	1.10	1.01	108-H	1.60	1.57	
	107-H	0.98	1.01	110-H	6.62	6.88	
	108-H	0.92	1.01				
	110-H	1.49	1.15				
	112-H	6.57	6.82				
	113-H	2.25	2.06				
	114-H	2.08	2.06				

Table S3. Theoretical and experimental values (cm^{-1}) of the IR vibrational frequencies of compounds **6**, **7**, **9**-**12** in vacuum, calculated using ωB97XD/6-311++G(2d,p).

Compd.	Theo.	Exp.	Assignments*	Compd.	Theo.	Exp.	Assignments
**6**	3679	3477	-OH(ν_s)		1096	1100	-CH$_2$(ρ)
	2953	2937	-CH$_2$(ν_{as})	**10**	3749	3491	-OH(ν_s)
	1790	1763	-C=O(ν_s)		3473	3433	-OH(ν_s)
	1344	1375	-CH$_3$(δ)		1754	1763	-C=O(ν_s)
	1241	1244	-CH$_2$(τ)		1745	1730	-C=O(ν_s)
	1189	1169	-CH$_2$(ρ)		1685	1609	-C=C(ν_s)
	1039	1049	-CH(ω)		1354	1368	-CH3(δ)
**7**	3748	3448	-OH(ν_s)		1232	1242	-CH2(τ)
	2954	2926	-CH$_2$(ν_{as})		1041	1035	-CH(ω)
	1790	1758	-C=O(ν_s)	**11**	3475	3432	-OH(ν_s)
	1736	/	-C=O(ν_s)		2938	2937	-CH$_2$(ν_{as})
	1665	1663	-C=C(ν_s)		1743	1734	-C=O(ν_s)
	1394	1379	-CH$_3$(δ)		1685	1610	-C=O(ν_s)
	1239	1253	-CH$_2$(τ)		1363	1364	-CH3(δ)
	1164	1167	-CH$_2$(ρ)	**12**	3741	3458	-OH(ν_s)
	1037	1027	-CH(ω)		2959	2931	-CH$_2$(ν_{as})
**9**	3729	3416	-OH(ν_s)		2893	2856	-CH$_2$(ν_{as})
	2982	2958	-CH$_2$(ν_{as})		1743	1734	-C=O(ν_s)
	1763	1729	-C=O(ν_s)		1684	1614	-C=C(ν_s)
	1687	1607	-C=C(ν_s)		1365	1364	-CH$_3$(δ)
	1456	1456	-CH(δ)		1244	1250	-CH$_2$(τ)

* ν_s: symmetrical stretching vibration; ν_{as}: asymmetric stretching vibration; ρ: planar rocking vibration; δ: shearing vibration; τ: torsional vibration; ω: out-of-plane rocking vibration

Table S4. Relevant values and proportions of molecular surface analysis for twelve compounds.

Compd.	Electrostatic potential (kcal·mol-1)	Area (Å2)	Proportion (%)	Compd.	Electrostatic potential (kcal·mol-1)	Area (Å2)	Proportion (%)
	-41.83	7.75	2.85		-41.83	6.99	1.50
	-35.50	8.44	3.10		-35.50	10.96	2.35
	-29.17	7.51	2.76		-29.17	16.92	3.63
	-22.83	9.05	3.33		-22.83	20.73	4.44
	-16.50	8.78	3.23		-16.50	17.64	3.78
	-10.17	10.68	3.92		-10.17	20.30	4.35
	-3.83	23.28	8.55		-3.83	36.66	7.86
**1**	2.50	49.23	18.08	**7**	2.50	75.57	16.20
	8.83	69.19	25.41		8.83	122.16	26.19
	15.17	61.95	22.76		15.17	79.29	17.00
	21.50	9.76	3.58		21.50	48.03	10.30
	27.83	2.10	0.77		27.83	5.62	1.21
	34.17	1.72	0.63		34.17	2.83	0.61
	40.50	1.64	0.60		40.50	1.88	0.40
	46.83	1.16	0.42		46.83	0.81	0.17
**Sum**		**272.26**	**100.00**	**Sum**		**466.36**	**100.00**
	-40.07	6.88	2.47		-41.83	6.82	1.25
	-34.20	7.44	2.68		-35.50	12.38	2.26
	-28.33	9.15	3.29		-29.17	21.48	3.92
	-22.47	9.44	3.40		-22.83	25.19	4.60
	-16.60	7.67	2.76		-16.50	23.94	4.38
	-10.73	7.78	2.80		-10.17	25.93	4.74
	-4.87	16.77	6.03		-3.83	36.26	6.63
**2**	1.00	40.10	14.42	**8**	2.50	95.39	17.43
	6.87	55.27	19.88		8.83	152.36	27.84
	12.73	68.00	24.46		15.17	86.85	15.87
	18.60	39.33	14.15		21.50	43.14	7.88
	24.47	6.36	2.29		27.83	11.27	2.06
	30.33	1.36	0.49		34.17	2.95	0.54
	36.20	1.39	0.50		40.50	2.12	0.39
	42.07	1.10	0.39		46.83	1.14	0.21
**Sum**		**278.03**	**100.00**	**Sum**		**547.22**	**100.00**
	-40.93	7.31	2.12		-37.33	6.73	1.15
	-34.80	8.14	2.36		-32.00	17.38	2.97
	-28.67	11.23	3.25		-26.67	26.30	4.49
	-22.53	15.01	4.34		-21.33	23.11	3.95
	-16.40	12.72	3.68		-16.00	20.79	3.55
	-10.27	15.93	4.61		-10.67	21.42	3.66
	-4.13	26.36	7.63		-5.33	31.17	5.32
**3**	2.00	40.53	11.73	**9**	0.00	64.26	10.97
	8.13	77.04	22.30		5.33	112.42	19.20
	14.27	89.18	25.82		10.67	135.98	23.22
	20.40	34.16	9.89		16.00	84.66	14.46
	26.53	3.72	1.08		21.33	24.91	4.25
	32.67	1.62	0.47		26.67	9.34	1.59
	38.80	1.38	0.40		32.00	3.72	0.63
	44.93	1.08	0.31		37.33	2.55	0.44

**Sum**		**345.41**	**100.00**	**Sum**		**584.72**	**99.86**
	-39.60	11.09	3.48		-46.67	8.23	1.23
	-34.80	12.86	4.03		-40.00	10.16	1.52
	-30.00	11.42	3.58		-33.33	14.93	2.24
	-25.20	9.69	3.04		-26.67	26.07	3.91
	-20.40	8.05	2.52		-20.00	32.55	4.88
	-15.60	7.07	2.22		-13.33	33.65	5.04
	-10.80	7.99	2.50		-6.67	47.41	7.10
**4**	-6.00	10.36	3.25	**10**	0.00	82.62	12.38
	-1.20	16.31	5.12		6.67	140.97	21.13
	3.60	34.02	10.67		13.33	146.93	22.02
	8.40	38.12	11.95		20.00	98.95	14.83
	13.20	60.24	18.89		26.67	17.60	2.64
	18.00	50.61	15.87		33.33	3.29	0.49
	22.80	32.90	10.32		40.00	3.05	0.46
	27.60	8.18	2.56		46.67	0.84	0.13
**Sum**		**318.90**	**100.00**	**Sum**		**667.25**	**100.00**
	-38.93	9.16	2.05		-46.33	7.63	1.23
	-32.80	16.76	3.76		-39.00	14.66	2.37
	-26.67	18.41	4.12		-31.67	21.64	3.50
	-20.53	18.46	4.14		-24.33	26.03	4.21
	-14.40	15.85	3.55		-17.00	28.93	4.68
	-8.27	18.67	4.18		-9.67	42.16	6.82
	-2.13	35.28	7.90		-2.33	75.03	12.13
**5**	4.00	53.20	11.92	**11**	5.00	133.57	21.60
	10.13	106.03	23.75		12.33	130.63	21.13
	16.27	90.18	20.20		19.67	104.28	16.87
	22.40	47.88	10.73		27.00	21.97	3.55
	28.53	12.07	2.71		34.33	4.86	0.79
	34.67	1.63	0.37		41.67	3.55	0.57
	40.80	1.38	0.31		49.00	2.02	0.33
	46.93	1.41	0.32		56.33	1.34	0.22
**Sum**		**446.37**	**100.00**	**Sum**		**618.29**	**100.00**
	-40.03	9.27	1.81		-44.57	8.82	1.24
	-34.10	12.80	2.51		-37.70	18.63	2.62
	-28.17	19.02	3.72		-30.83	24.57	3.46
	-22.23	20.93	4.10		-23.97	33.59	4.73
	-16.30	17.46	3.42		-17.10	33.81	4.76
	-10.37	21.71	4.25		-10.23	43.95	6.18
	-4.43	38.61	7.56		-3.37	98.78	13.90
**6**	1.50	63.56	12.44	**12**	3.50	179.69	25.28
	7.43	119.27	23.35		10.37	155.30	21.85
	13.37	113.91	22.30		17.23	81.64	11.49
	19.30	64.27	12.58		24.10	18.02	2.54
	25.23	5.86	1.15		30.97	5.30	0.75
	31.17	1.61	0.32		37.83	4.28	0.60
	37.10	1.35	0.26		44.70	2.88	0.41
	43.03	1.27	0.25		51.57	1.54	0.22
**Sum**		**510.90**	**100.00**	**Sum**		**710.81**	**100.00**

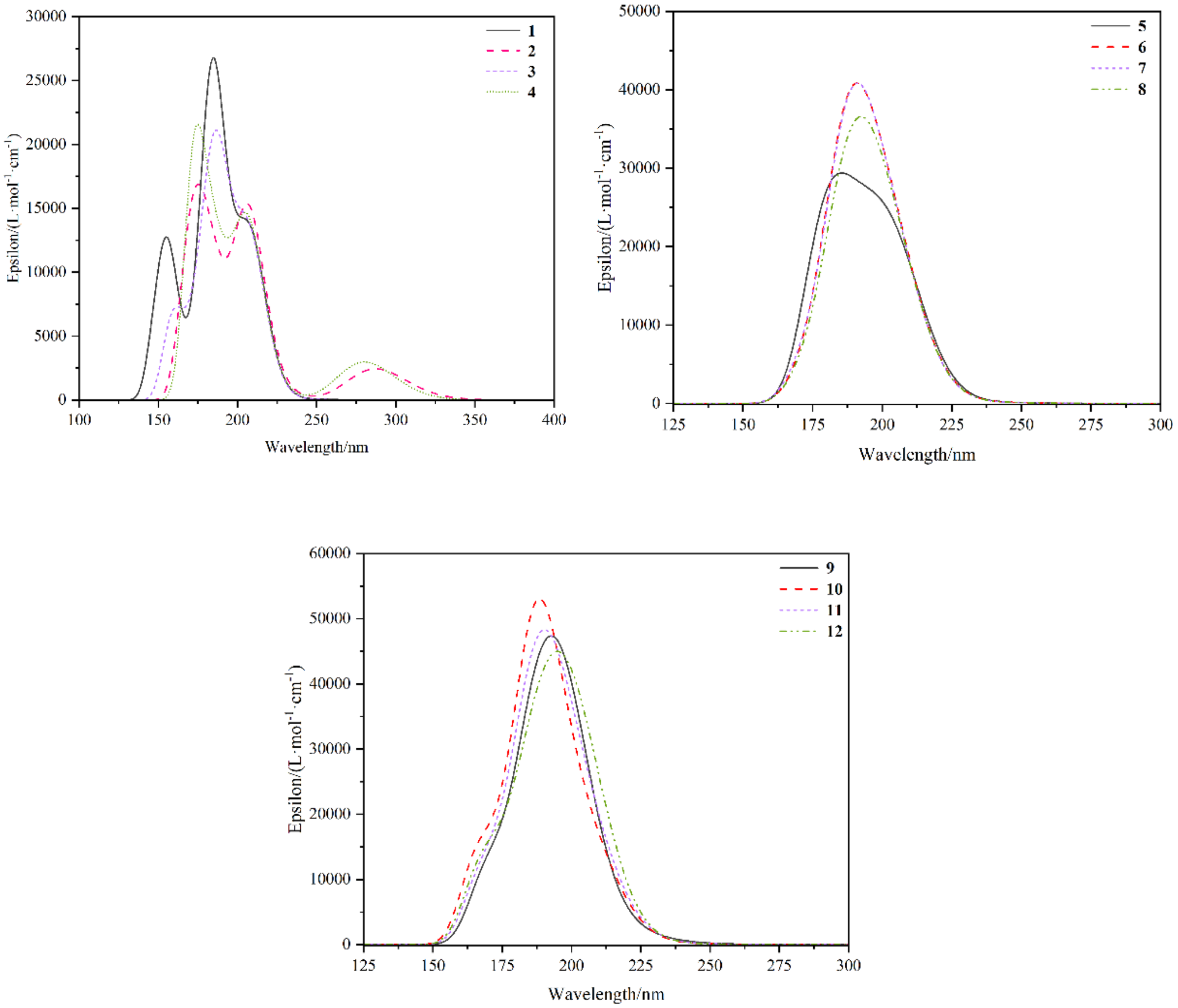

Figure S1. UV-Vis absorption spectra of twelve compounds in methanol calculated using ωB97XD/6-311++G(2d,p) method.

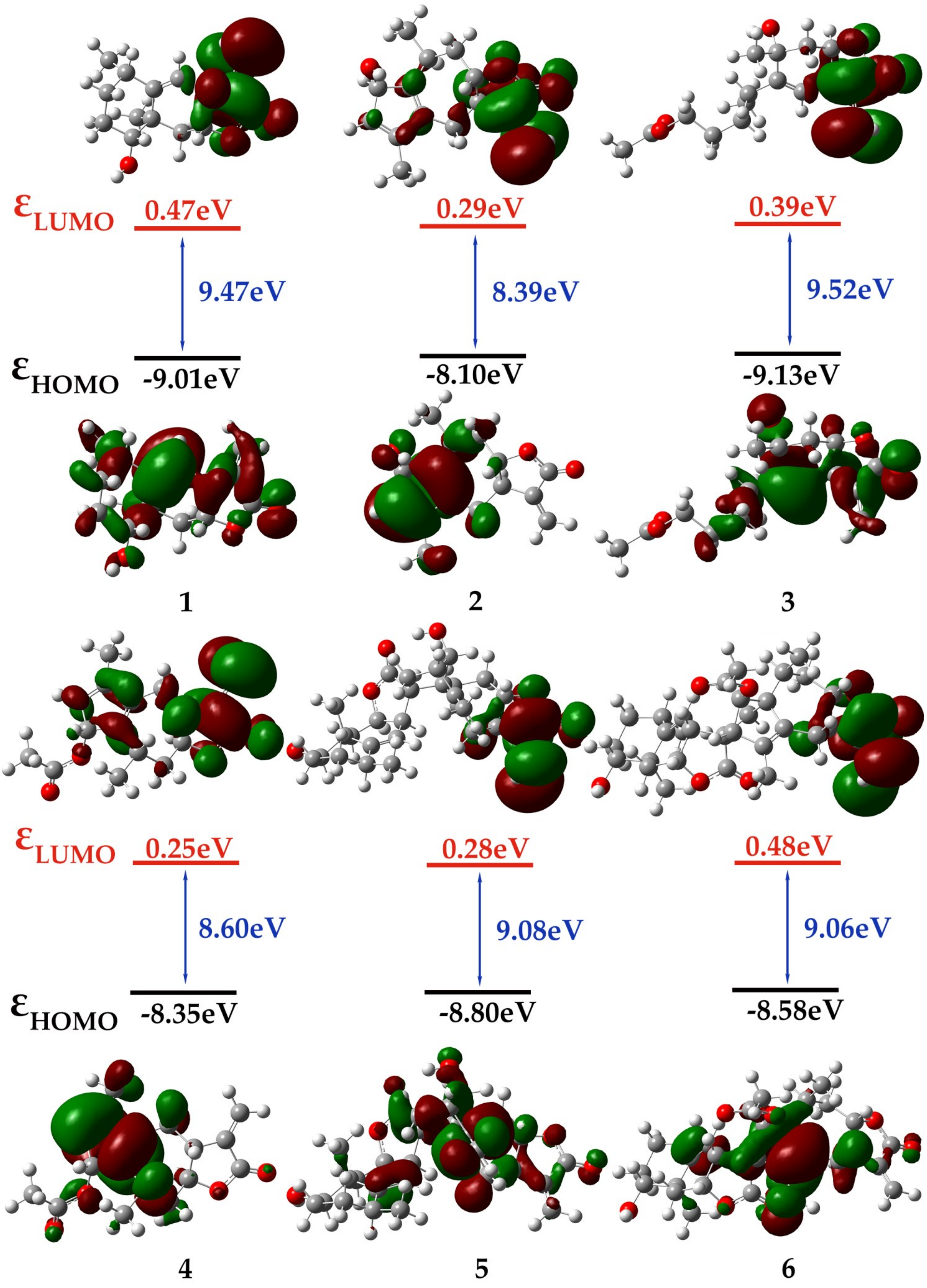

ε LUMO
0.47eV
0.29eV
0.39eV
9.47eV
8.39eV
9.52eV
ε HOMO
-9.01eV
-8.10eV
-9.13eV
1
2
3
ε LUMO
0.25eV
0.28eV
0.48eV
8.60eV
9.08eV
9.06eV
ε HOMO
-8.35eV
-8.80eV
-8.58eV
4
5
6

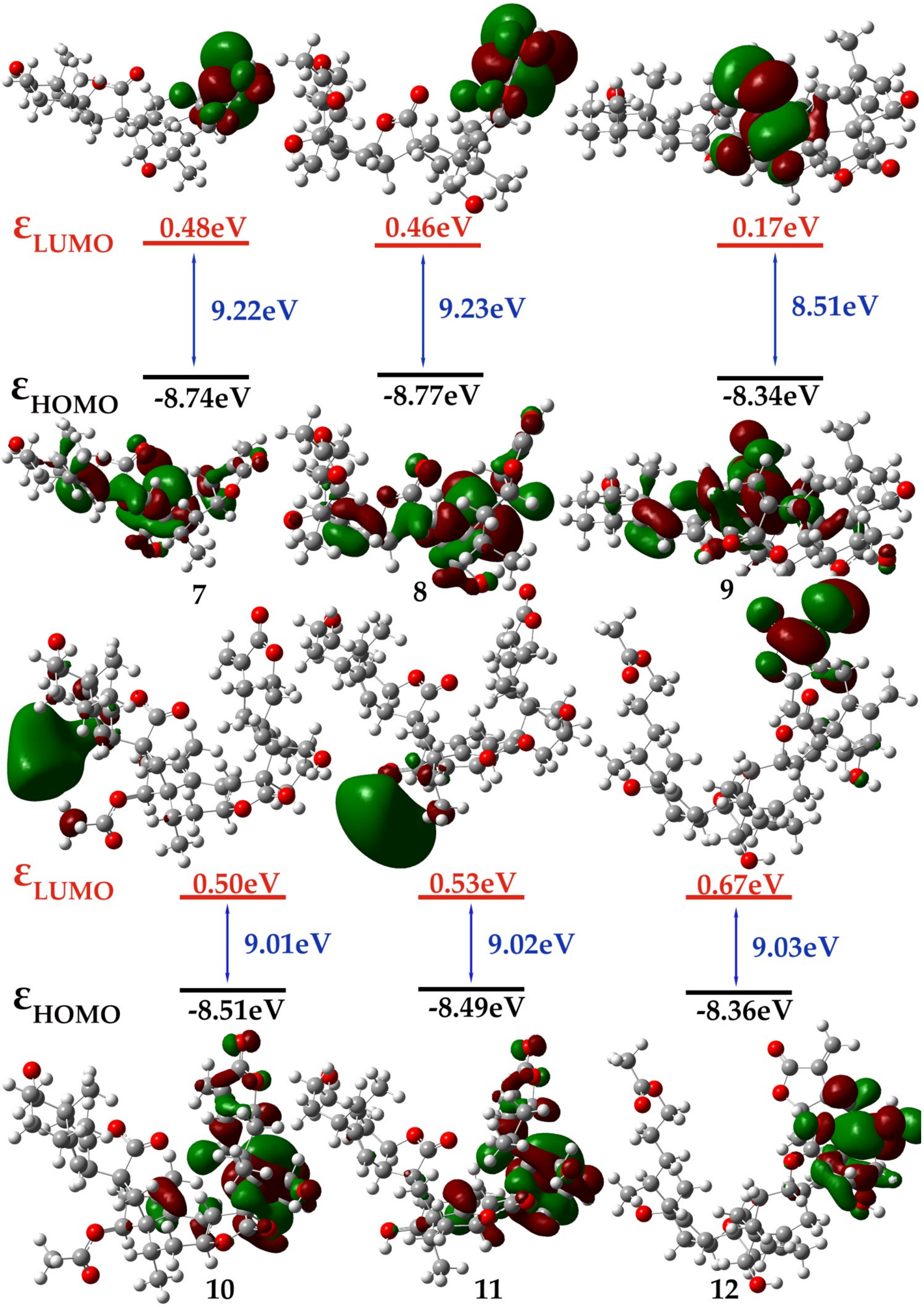

Figure S2. Frontline molecular orbital diagrams of twelve compounds in a vacuum environment.

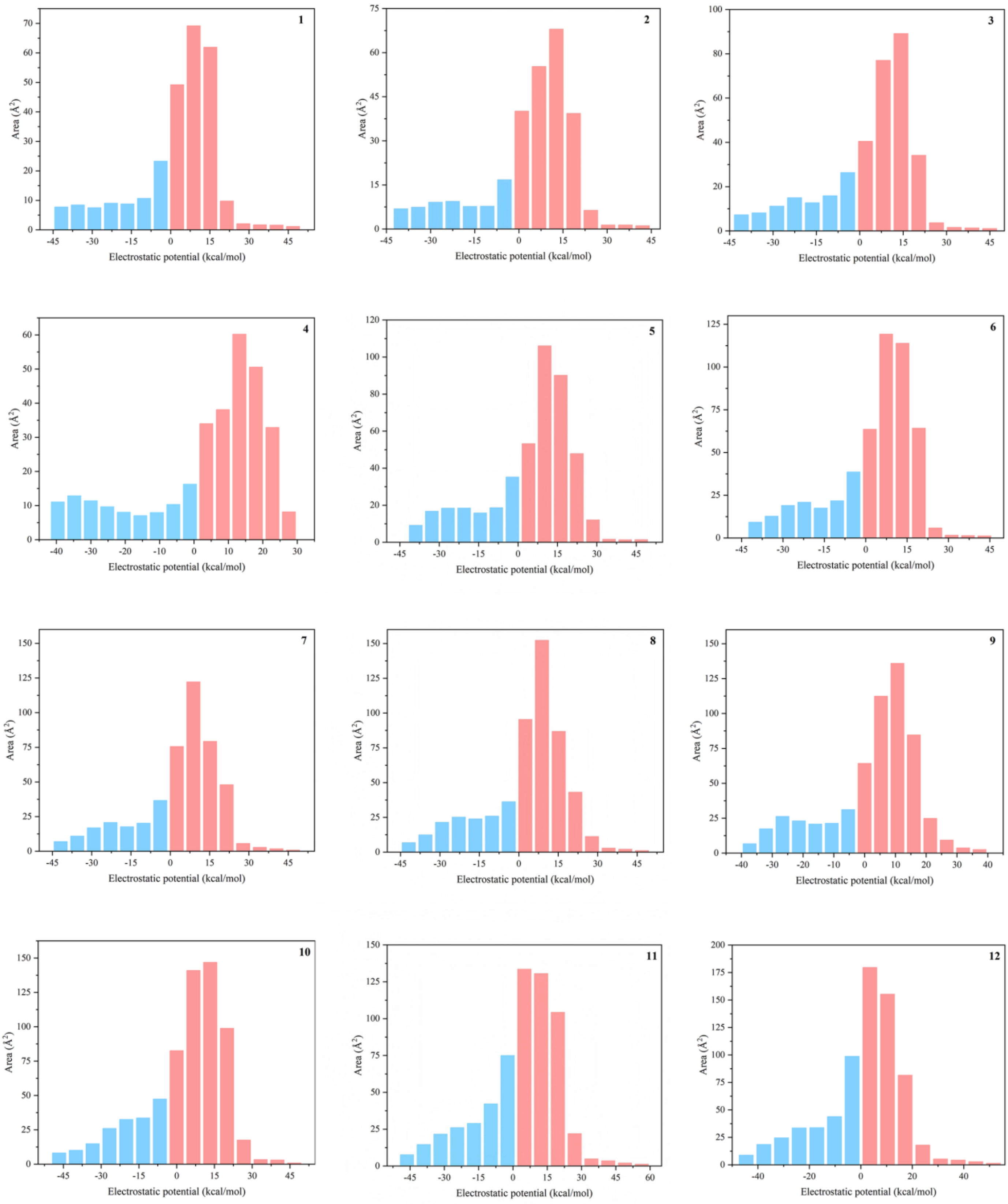

Figure S3. Surface area distribution diagram of the electrostatic potential interval of the compounds in a vacuum.

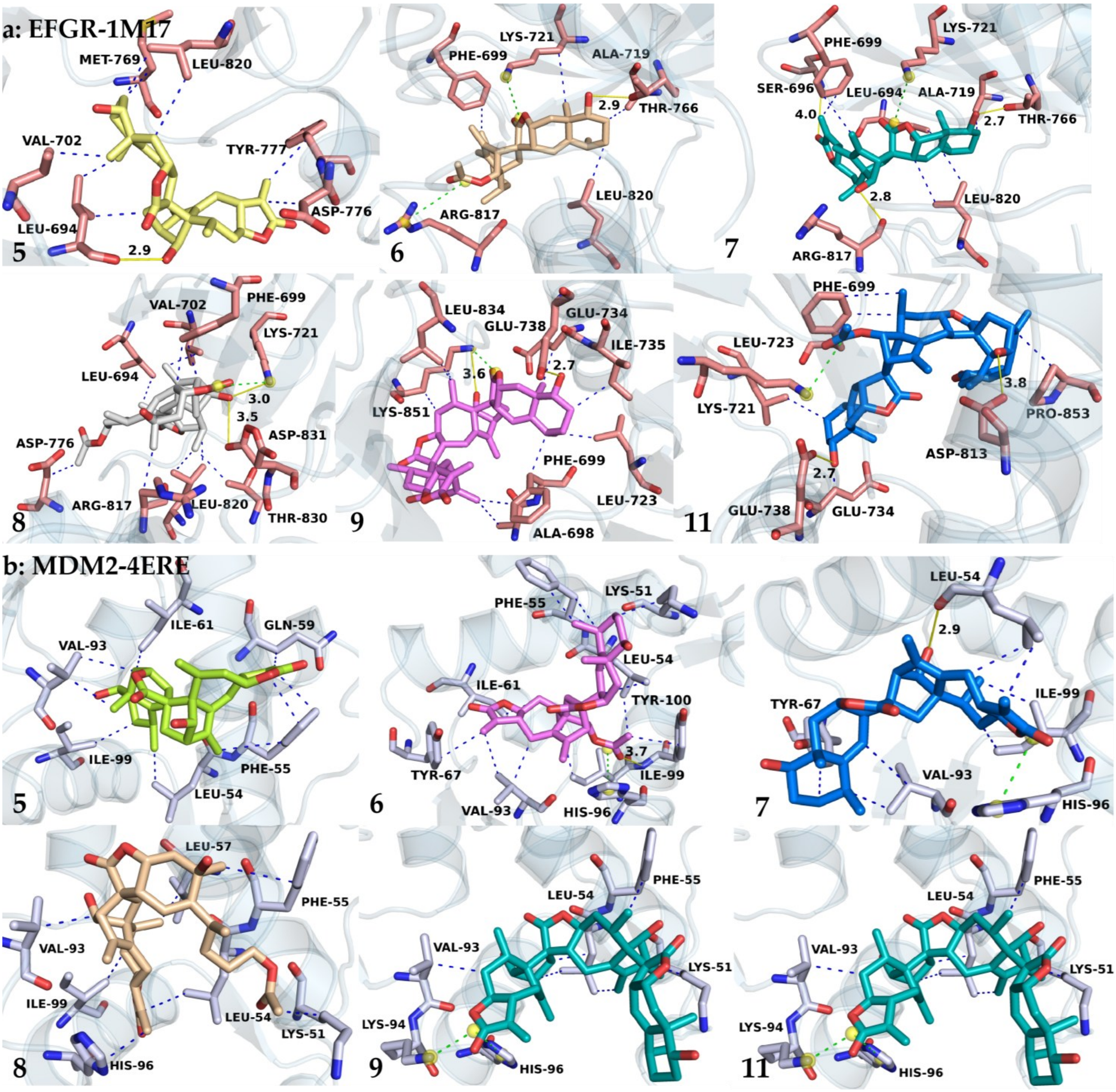

a: EFGR-1M17
MET-769
LEU-820
VAL-702
TYR-777
ASP-776
LEU-694
2.9
5
PHE-699
LYS-721
ALA-719
2.9
THR-766
LEU-820
ARG-817
6
PHE-699
LYS-721
SER-696
LEU-694
ALA-719
4.0
2.7
THR-766
2.8
LEU-820
ARG-817
7
VAL-702
PHE-699
LYS-721
LEU-694
3.0
3.5
ASP-776
ASP-831
ARG-817
LEU-820
THR-830
8
LEU-834
GLU-738
GLU-734
ILE-735
2.7
3.6
LYS-851
PHE-699
LEU-723
ALA-698
9
PHE-699
LEU-723
LYS-721
3.8
PRO-853
ASP-813
2.7
GLU-738
GLU-734
11
b: MDM2-4ERE
ILE-61
GLN-59
VAL-93
ILE-99
PHE-55
LEU-54
5
PHE-55
LYS-51
LEU-54
ILE-61
TYR-100
TYR-67
3.7
ILE-99
VAL-93
HIS-96
6
LEU-54
2.9
TYR-67
ILE-99
VAL-93
HIS-96
7
LEU-57
PHE-55
VAL-93
ILE-99
LEU-54
LYS-51
HIS-96
8
PHE-55
LEU-54
VAL-93
LYS-51
LYS-94
HIS-96
9
PHE-55
LEU-54
VAL-93
LYS-51
LYS-94
HIS-96
11

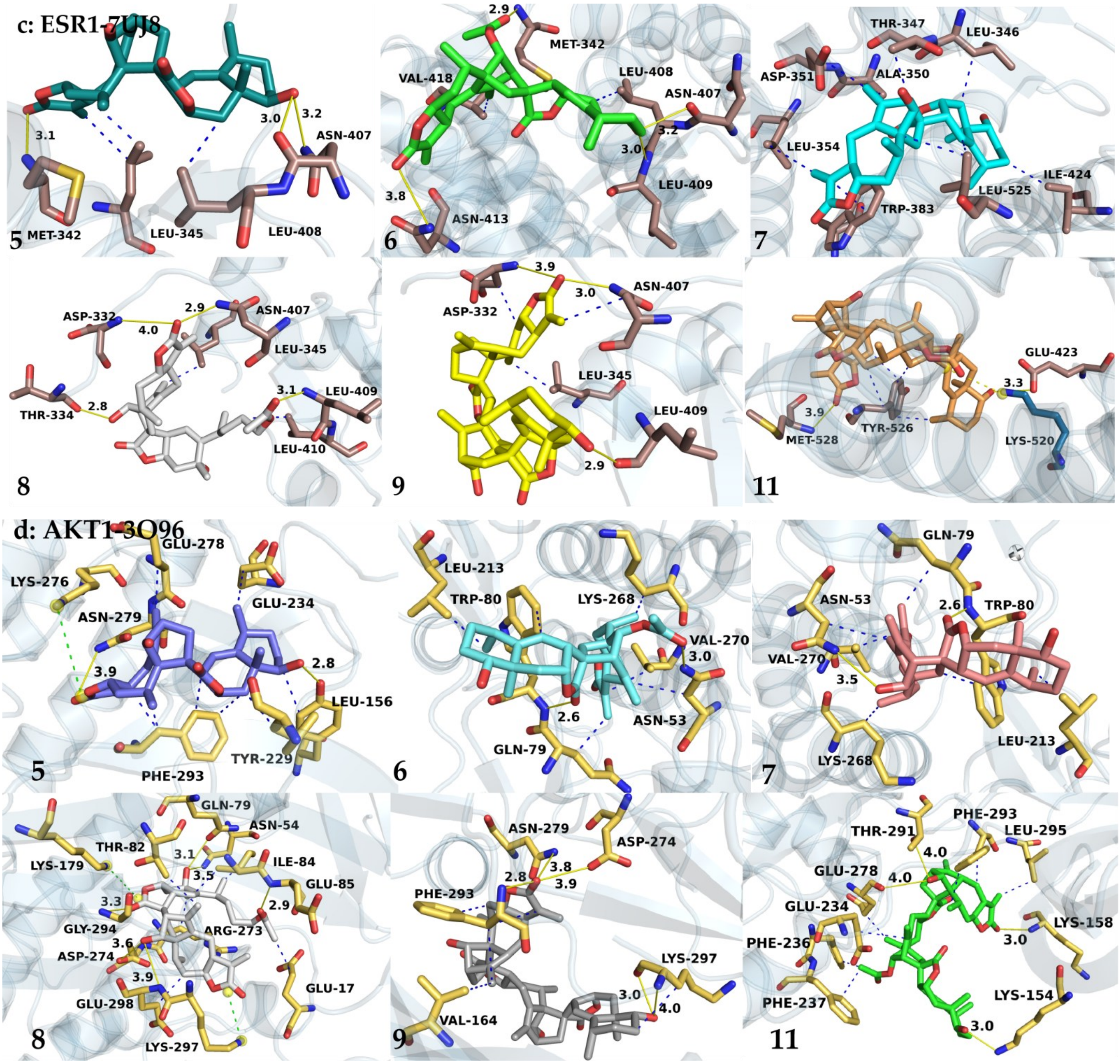

c: ESR1-7UJ8
3.1
3.0
3.2
ASN-407
MET-342
LEU-345
LEU-408
5
2.9
MET-342
VAL-418
LEU-408
ASN-407
3.2
3.0
LEU-409
3.8
ASN-413
6
THR-347
LEU-346
ASP-351
ALA-350
LEU-354
ILE-424
LEU-525
TRP-383
7
ASP-332
2.9
ASN-407
4.0
LEU-345
THR-334
2.8
3.1
LEU-409
LEU-410
8
3.9
ASN-407
3.0
ASP-332
LEU-345
LEU-409
2.9
9
GLU-423
3.3
3.9
MET-528
TYR-526
LYS-520
11
d: AKT1-3O96
GLU-278
LYS-276
GLU-234
ASN-279
2.8
3.9
LEU-156
PHE-293
TYR-229
5
LEU-213
TRP-80
LYS-268
VAL-270
3.0
2.6
ASN-53
GLN-79
6
GLN-79
ASN-53
2.6
TRP-80
VAL-270
3.5
LEU-213
LYS-268
7
GLN-79
ASN-54
THR-82
3.1
ILE-84
LYS-179
3.5
GLU-85
3.3
2.9
GLY-294
ARG-273
3.6
ASP-274
GLU-17
3.9
GLU-298
LYS-297
8
ASN-279
ASP-274
3.8
2.8
PHE-293
3.9
LYS-297
3.0
4.0
VAL-164
9
THR-291
PHE-293
LEU-295
4.0
GLU-278
4.0
GLU-234
LYS-158
3.0
PHE-236
PHE-237
LYS-154
3.0
11

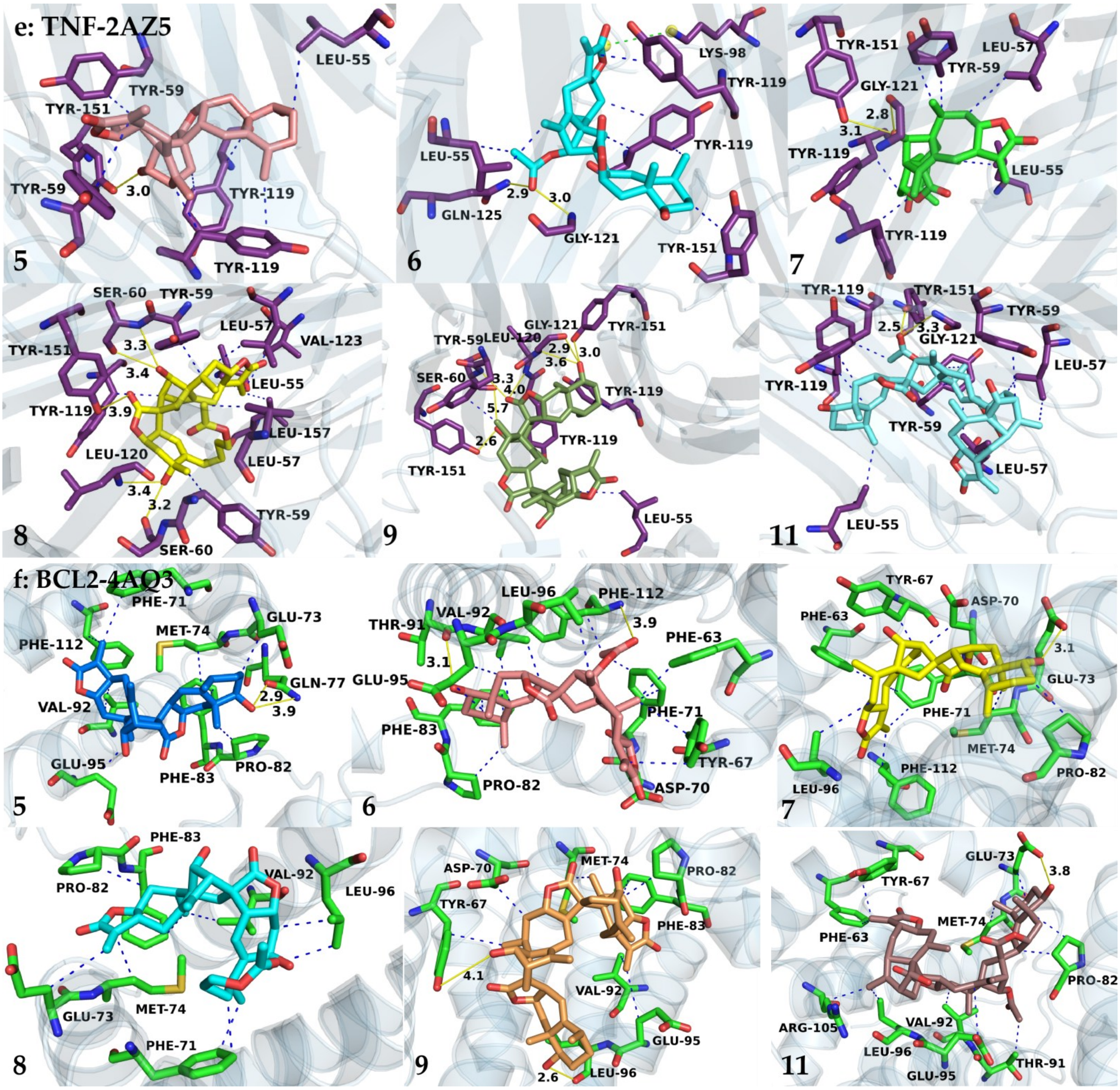

e: TNF-2AZ5
LEU-55
TYR-59
TYR-151
TYR-59
3.0
TYR-119
TYR-119
5
LYS-98
TYR-119
LEU-55
TYR-119
2.9
3.0
GLN-125
GLY-121
TYR-151
6
TYR-151
LEU-57
TYR-59
GLY-121
2.8
3.1
TYR-119
LEU-55
TYR-119
7
SER-60
TYR-59
LEU-57
VAL-123
3.3
TYR-151
3.4
LEU-55
TYR-119
3.9
LEU-157
LEU-120
LEU-57
3.4
3.2
TYR-59
SER-60
8
GLY-121
TYR-151
TYR-59
LEU-120
2.9
3.6
3.0
SER-60
3.3
4.0
TYR-119
5.7
2.6
TYR-119
TYR-151
LEU-55
9
TYR-119
TYR-151
TYR-59
2.5
3.3
GLY-121
LEU-57
TYR-119
TYR-59
LEU-57
LEU-55
11
f: BCL2-4AQ3
PHE-71
GLU-73
MET-74
PHE-112
GLN-77
2.9
VAL-92
3.9
GLU-95
PHE-83
PRO-82
5
LEU-96
PHE-112
THR-91
VAL-92
3.9
PHE-63
3.1
GLU-95
PHE-71
PHE-83
TYR-67
PRO-82
ASP-70
6
TYR-67
ASP-70
PHE-63
3.1
GLU-73
PHE-71
MET-74
PHE-112
LEU-96
PRO-82
7
PHE-83
VAL-92
PRO-82
LEU-96
GLU-73
MET-74
PHE-71
8
MET-74
ASP-70
PRO-82
TYR-67
PHE-83
4.1
VAL-92
GLU-95
2.6
LEU-96
9
GLU-73
3.8
TYR-67
MET-74
PHE-63
PRO-82
ARG-105
VAL-92
LEU-96
THR-91
GLU-95
11

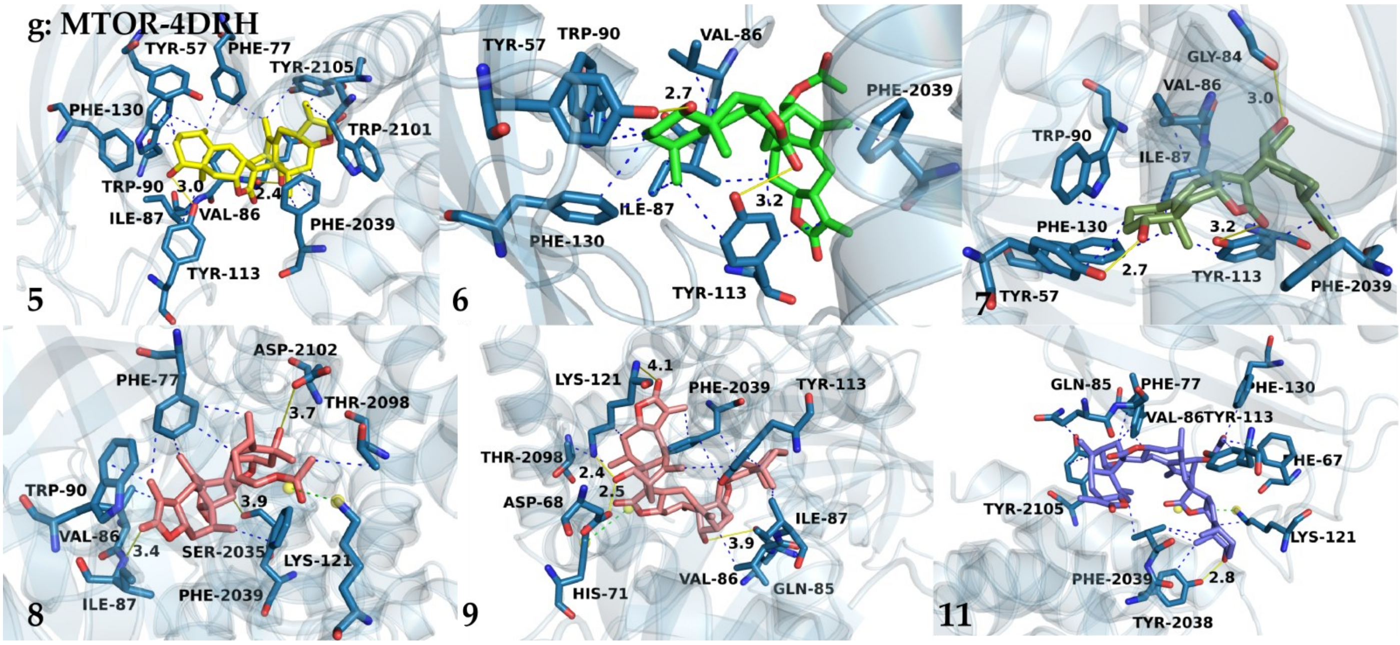

Figure S4. Molecular docking diagrams of compounds **5-9** and **11** with targets EGFR, MDM2, EXR1, TNF, BCL2, MTOR, and AKT1.
